# Diffusion meets edge awareness: a unified framework for high-precision ultrasound nerve segmentation

**DOI:** 10.3389/fdgth.2026.1802954

**Published:** 2026-07-09

**Authors:** Tanaya Mahalanabis, OmKumar Chandra Umakanthan

**Affiliations:** School of Computer Science and Engineering, Vellore Institute of Technology, Chennai, India

**Keywords:** attention U-Net, diffusion-based denoising, edge fusion, multi-channel feature fusion, ultrasound image segmentation

## Abstract

**Introduction:**

Nerve segmentation from ultrasound images is still challenging due to speckle noise, poor contrast, and unclear anatomical edges. These limitations have an adverse effect on deep learning segmentation models working on raw ultrasound data.

**Methods:**

In order to overcome these difficulties, a diffusion-guided edge-aware multi-channel segmentation approach that can take advantage of raw ultrasound images, diffusion-processed features, and edge information is proposed. Moreover, a new EdgeFusion-U-Net model is developed for integrating these complementary features. Experiments were performed on two benchmark datasets of ultrasound nerve segmentation and the proposed method was compared with other state-of-the-art techniques such as U-Net, Residual U-Net, UNet++, TransU-Net, nnU-Net, and Attention U-Net.

**Results:**

As evidenced by experiments, the proposed method shows better results than existing techniques. Our method reached Dice scores of 94.62% and 93.91% on two benchmark datasets, while baseline methods had about 83% and 84% Dice scores without applying diffusion denoising and edge fusion.

**Conclusion:**

It can be seen that incorporating diffusion filter-based speckle noise removal and edge-based feature fusion techniques helps to achieve better results in terms of boundary detection. This technique not only helps in obtaining accurate information about the nerves but can also be used to support computer-assisted clinical analysis using ultrasounds.

## 1 Introduction

Ultrasound imaging is now an important technique used in contemporary clinical applications because of its non-invasive characteristics, real-time visualization features, and economical nature. The use of ultrasound in imaging procedures is diverse and includes procedures like anaesthesia, trauma care, and musculoskeletal assessments. The process of locating and identifying the position of peripheral nerves is one of the significant uses of ultrasound imaging technology.

On the other hand, the process of identifying peripheral nerves using ultrasound imaging is a complicated procedure. Nerves are often very small and have a complex morphology. They do not have a distinct appearance compared to other body parts like bones or organs and hence cannot be easily identified by visualizing their shape. Moreover, the ultrasound images themselves contain several defects such as intensity inhomogeneities, blurred edges, and speckle noise. In addition, ultrasound scans require a high level of expertise from the operator to produce high-quality images that can be interpreted effectively.

Correct segmentation of nerves is important for many clinical applications, especially in cases involving post-operative pain control. Previously, opioids were commonly prescribed to patients undergoing surgery to relieve their pain. Although this treatment was successful, it came with various side effects, such as vomiting, drowsiness, delayed recovery time, and the possibility of developing addiction. As a result, regional anaesthesia methods, which involved administering anaesthetics close to the desired nerves via catheters, have become increasingly popular. Such methods depend largely on accurate segmentation of nerves from ultrasound images, which would prevent inadequate treatment or injury to nearby tissues otherwise.

Nerve segmentation plays a significant role in emergency medicine and injury treatment settings. Since injuries that occur as a result of an accident frequently affect soft tissues and nerves, these structures can hardly be identified using conventional methods, such as radiographic techniques like x-ray and computed tomography scans. Thus, one possible solution involves ultrasound-based assessments. While such an imaging technique is relatively accessible and convenient, identifying specific nerve structures through it proves to be complicated due to poor imaging quality. The implementation of automated segmentation solutions will be useful in facilitating the process of identification of nerve structures.

A great variety of modern architectures of deep neural networks used for segmentation tasks have proven their efficiency. U-Net, residual U-Net, and attention U-Net models are just some of the examples of highly efficient deep neural network models. Nevertheless, when it comes to ultrasound images, current architectures show several weaknesses. First, speckle noise can prevent the identification of fine structures. Second, the boundary is ambiguous and may cause the leakage of nerve structures into adjacent tissues. Third, a low level of contrast makes it difficult to detect nerve structures within the ultrasound image.

A number of research works aimed at tackling these issues using both model-based and data-driven methods. Pratisthitha et al. (1) suggested a probabilistic deep learning technique with Bayesian inference accounting for uncertainties in segmenting the nerves, yet it exhibits considerable complexity and poor scalability. Kumar et al. (2) suggested using ResU-Net architecture to improve feature transfer within the neural network, yet the model is susceptible to noise. Mori et al. (3) investigated the role played by different resolutions in image scaling and processing, yet they did not take robustness into account. Wang et al. (4) suggested a force-sensing driven segmentation technique, which improves anatomical accuracy of nerve segmentation, but it uses auxiliary hardware components. Peng et al. (5) developed a method for visualizing neck nerves based on ultrasound images, which gives us qualitative insights, yet it lacks an automation aspect.

Still, there is a problem of designing robust segmentation techniques for ultrasounds, since none of the suggested solutions can deal with the issues associated with noise, boundary enhancement, and feature representation.

The main idea of this research is about creating a tool for looking at nerves in ultrasound pictures. This tool is called a Lightweight Diffusion-Guided Edge-Enhanced Fusion Framework for Ultrasound Nerve Segmentation.

1. The thing about this method is that it uses a way to get rid of the noise in ultrasound pictures called lightweight diffusion-based speckle denoising. This method of diffusion-based speckle denoising is really good at removing the noise, from ultrasound images without hurting the tiny details that doctors need to see. The best part is that this method of lightweight diffusion-based speckle denoising works well even on old computers that are not very powerful.
2. When we look at the edges of things we can find the borders of nerves. This is called edge- boundary extraction. It helps computers find the right parts of nerves to look at. The computers use tools to find the thin and hard to see borders of nerves. This guides the computers to the parts of the nerves that're important to understand. The computers use something called segmentation networks to do this. These networks help the computers find the parts of the nerves. Edge-aware boundary extraction is important for this to work. It helps the computers find the edges of nerves so they can look at the parts.
3. We have three kinds of images: the image, the image with noise removed and the image, with the edges made clearer. When we combine these three images, we get an image that has three channels. This new image is really good because it keeps the context is clear. Has sharp boundaries. The tri-modal fusion of images denoised images and edge-enhanced images is what makes this happen. The fusion block is the part of the proposed pipeline. It helps to make the features cleaner and more useful. These features are really good for segmentation. They are better than what you get from models that only use one input. The fusion block makes a difference, in how accurate the segmentation is. It is very important for the proposed pipeline to work well. The fusion block is what makes the proposed pipeline special.

The key contributions of this research can be listed as follows:

- Diffusion-steered ultrasound denoising for enhanced feature extraction: A diffusion-based denoising module is added as a preprocessing step to efficiently remove speckle noise and retain key anatomical details in ultrasound images.
- Edge-aware multi-channel feature fusion via the proposed EdgeFusion module: An edge-informed feature extraction process is proposed to extract boundary structural information, which is then fused with the denoised ultrasound images using a specialized EdgeFusion module, allowing the network to learn both texture and boundary features.
- A comprehensive segmentation solution for robust ultrasound nerve image segmentation: The proposed EdgeFusion-U-Net model combines diffusion denoising, edge-aware feature enhancement, and Attention U-Net-based segmentation into a single solution, enhancing segmentation performance on a variety of ultrasound nerve image datasets.

Through the integration of diffusion-driven denoising, boundary-aware enhancement, and multi-modal fusion, the proposed framework addresses long-standing challenges in ultrasound nerve segmentation. The resulting system delivers sharper anatomical representations and more reliable nerve localization, paving the way for safer pain-management procedures, improved trauma-care diagnostics, and scalable clinical deployment in resource-limited healthcare settings. The subsequent sections present the methodology, experimental setup, and comprehensive performance analysis of the proposed framework.

## 2 Related works

Although ultrasound-based nerve segmentation has gradually advanced from traditional image-processing pipelines to contemporary deep learning architectures, speckle noise, low contrast, and irregular nerve boundaries continue to make the problem difficult. The majority of current techniques mainly rely on single raw ultrasound inputs without specifically addressing speckle suppression or boundary-focused feature enhancement, despite recent efforts to increase segmentation accuracy through architectural innovations and contextual modelling.

Pradhiba et al. (6) proposed a RESU-Net variant for real-time ultrasound segmentation of the TP block region and showed that encoder-decoder refinements can support clinicians during live scanning. While such a refinement helped to improve operational feasibility, their study did not involve explicit denoising or edge enhancement mechanisms and therefore robustness to operator variability and generalization were not adequately addressed. Similarly, Wyss et al. (7) conducted a systematic evaluation with respect to clinical nerve segmentation relevance of standard segmentation metrics and identified that most of the standard quantitative metrics often do not agree with clinical interpretability. Their work brings out the need for standardized evaluation protocols but does not present methodological propositions towards better segmentation performance.

There is also temporal and contextual cue exploration. Xiang Li et al. (8) proposed an artery/vein segmentation framework using force-sensing sequential ultrasound, which demonstrated temporal continuity and sensor feedback can enhance vascular segmentation. However, this work was targeted on vascular structures with no nerve-specific boundary ambiguities and speckle-noise suppression. Similar to the discussed approach, Watanabe et al. (9) also systematically studied network depth, width, and image scaling for nerve detection, concluding that architectural scaling significantly affects the accuracy of the detection. At the same time, this work remains in the circle of network sizing without touching input enhancement or explicit noise handling.

Previous deep learning techniques for ultrasound nerve analysis mostly emphasized network architecture rather than conditioning of the data/images. Biswas et al. (10) proposed a deep learning framework called DeepNerve for median nerve localization and segmentation in ultrasound sequences, showing benefits in sequence processing. Although promising in ultrasound sequence processing for nerve segment detection, this method only focused on median nerve detection without incorporating multimodal data or noise elimination techniques. Wu et al. (11) also proposed a model called a “Region-aware Global Context Modeling Network” to enhance the detection of ill-described nerve regions based on the incorporation of contextual dependencies in the process of recognition. This model also lacks the process of noise elimination based on the principle of diffusion or fusion of feature edges. Casaletto et al. (12) and Huang et al. (13) also conducted clinical correlation studies to prove the usability of ultrasound for nerve detection as well as automated detection of femoral nerve blocks, respectively.

Probabilistic approaches within probabilistic and uncertainty-aware models were also considered by Iresha Rubasinghe and Meedeniya (14), probabilistic programming, incorporating U-Net architectures along with several types of GANs, to approach estimation of uncertainties within segmentation tasks. Despite the promises that probabilistic and uncertainty-aware approaches hold, the framework did not explore or make use of diffusion speckle removal or edge fusion, as seen within other architectures. Nwawka et al. (15) presented a review article focused around high-resolution approaches for imaging a peripheral nervous system through an ultrasound imaging technique; despite this, it did not explore or make use of any automatic segmentation approach as seen within other architectures, incorporating more imaging baselines within their work. In one of the first automatic approaches toward the segmentation of a nerve, Baby and Jeresh (16) utilized classical ML, along with several types of early CNN architectures, despite limited data and other constraints.

Hafiane et al. studied spatio-temporal consistency and active contour integration in spatiotemporal image analysis in detail in (17). They used an integration of CNN-based localization and temporal information and models to enhance robustness. Nevertheless, the contribution of this paper was not denoising and integration of multi-representative information. Hadjerci et al. and González et al. presented comparative studies in (18) and (19), respectively. Classical approaches to nerve localization used features of images and wavelet transforms in addition to graph-cut segmentation. Their approaches worked satisfactorily but were heavily affected by speckle noise. Their models were not suitable and did not generalize well to current datasets. Sun et al. presented a coherent overview of the improvements brought by deep learning methods in the localization of nerves in ultrasound images in (20). Nevertheless, there were many inaccuracies in image datasets and evaluation methods.

However, other recent deep learning variants, such as the U2-Net by Kaya et al. (21), showed improved boundary preservation for median nerve segmentation, while still depending on single raw ultrasound inputs and without any additional denoising or fusion strategies. Li et al. (22) explored attention-based networks that incorporate attention mechanisms with VGG16 encoders to improve nerve localization. These models do not explicitly suppress speckle noise or incorporate multi-input fusion. Li et al. (23) proposed a nested attention for sharpening contours. However, this study focused on pure boundary modelling and did not integrate learned denoising or raw-denoised-edge input fusion. GAN-based adversarial segmentation was proposed by Doré et al. (24) but failed to improve segmentation plausibility while not addressing speckle noise removal or structured edge guidance.

Over the past few years, various innovative segmentation models have been proposed in the extensive medical image analysis literature, focusing on context-based learning and preserving object boundaries. For example, in the work by Zhang et al., titled ESKNet (25), which was published in Expert Systems with Applications (2024), an advanced selective kernel technique was used to enable adaptive feature learning at different scales, thus enhancing the quality of the segmentation process irrespective of the scale of objects. In another study, Liu et al. in their paper titled RRCNet (26) (Engineering Applications of Artificial Intelligence, 2023) used residual refinement and context aggregation techniques.

However, in two decades of literature on this problem, all studies focus on any of these architectural extensions, context modelling, SN consistency, or attention modelling. Only a handful of studies have explored the problem of ultrasound speckle reduction, nerve boundary sharpening, and multi-representational input fusion collectively in a unified manner. Thus, addressing this identified research gap constitutes the motivation for this work on proposing the framework of Diffusion Guided, Edge Enhanced Multi Representation Fusion. Table 1 Further summarizes the entire literature review.

**Table 1:** Existing works analysis.

Author & references	Technique used	Merits	Demerits	Dataset used
Pradhiba et al. (6)	RESU-NET (U-Net variant)	Real-time ultrasound segmentation for TP block region to assist clinicians	No explicit denoising or edge-enhancement; limited generalization analysis	Clinical ultrasound images (TP block region)
Wyss et al. (7)	Systematic evaluation/metric analysis	Assessed clinical relevance of segmentation metrics for nerve segmentation	Lack of standardized evaluation criteria; no algorithmic innovation	Multiple clinical ultrasound datasets
Xiang Li et al. (8)	Force-sensing sequential ultrasound model	Temporal and sensor-guided segmentation for artery/vein structures	Targets vessels, not nerves; no diffusion or edge fusion	Sequential ultrasound with force-sensing data
Toshiaki Watanabe et al. (9)	CNN scaling experiments	Studied effect of network and image scaling on nerve detection	Focused only on architecture scaling; no input enhancement	Ultrasound nerve datasets
Biswas et al. (10)	DeepNerve CNN	Median nerve localization and segmentation in ultrasound sequences	Limited to median nerve; lacks multi-input fusion or denoising	Median nerve ultrasound sequences
Huisi Wu et al. (11)	Region-aware global context modeling	Improved segmentation of blurred and low-contrast nerves	No explicit denoising or edge-guided fusion	Ultrasound nerve images
Casaletto et al. (12)	Clinical ultrasound correlation study	Validated ultrasound visualization of neck nerves	Clinical study only; no automated segmentation	Clinical neck ultrasound scans
Huang et al. (13)	Deep learning for femoral nerve block recognition	Demonstrated automation potential in nerve block procedures	Focused on femoral nerve; no denoising or fusion	Femoral nerve ultrasound images
Rubasinghe & Meedeniya (14)	Probabilistic programming + U-Net + GAN	Uncertainty-aware nerve segmentation models	No diffusion-based denoising or explicit edge fusion	Ultrasound nerve datasets
Nwawka et al. (15)	High-resolution ultrasound imaging review	Provided imaging baselines for nerve visualization	Descriptive review; no ML-based segmentation	Multiple clinical ultrasound studies
Baby & Jeresh (16)	Classical ML/early CNN pipeline	Early attempt at automatic nerve segmentation	Limited datasets; outdated architectures	Small-scale ultrasound datasets
Hafiane et al. (17)	CNN + spatio-temporal consistency + contours	Enforced temporal consistency in ultrasound segmentation	No explicit denoising or fusion framework	Temporal ultrasound sequences
Hadjerci et al. (18)	Comparative CAD study	Compared classical ML approaches for nerve detection	Pre-deep learning; poor speckle noise handling	Ultrasound nerve images
González et al. (19)	Graph-cut + wavelet + GP classifiers	Structured classical pipeline for nerve segmentation	Handcrafted features; weak generalization on noisy images	Ultrasound nerve datasets
Sun et al. (20)	Systematic deep learning review	Highlighted advantages of DL for nerve localization	Dataset and metric inconsistency; no method proposed	Multiple reported datasets
Elif Kaya et al. (21)	U²-Net variant	Boundary-preserving median nerve segmentation	Single raw input only; no fusion or denoising	Median nerve ultrasound images
Li et al. (22)	Attention + VGG16 + U-Net	Attention-enhanced nerve segmentation	No explicit denoising or multi-input fusion	Ultrasound nerve datasets
Li et al. (23)	Nested attention + boundary guidance	Sharpened nerve contour segmentation	Boundary modeling only; no diffusion denoising	Ultrasound nerve images
Doré et al. (24)	GAN-based segmentation	Improved segmentation plausibility via adversarial learning	No explicit speckle noise handling or edge fusion	Ultrasound nerve datasets
Shiyu Zhang et al. (25)	Dataset creation	Provided high-resolution ultrasound datasets for benchmarking	No segmentation methodology proposed	High-resolution ultrasound datasets
Chen et al. (26)	C-Net: Context-Aware/Complementary Local-Global Feature Learning Network for Medical Image Segmentation	Provides better context representation, maintains boundary details, and increases segmentation accuracy by integrating information from both local and global features	Not designed to handle the specific artifacts of ultrasound imaging, such as speckle noise, low contrast, and abnormal nerve boundaries	Medical image datasets

### 2.1 Problem statement

The issue of segmenting ultrasound images continues to pose a significant challenge for several reasons. Firstly, ultrasound imaging inherently produces images that suffer from speckle noise, low contrast, and blurred anatomical boundaries. This severely limits the ability to detect and delineate nerves. Additionally, variations in image quality owing to different image acquisition parameters and operator proficiency levels compound the problem. Despite the impressive results reported by CNNs such as U-Net in performing medical image segmentation tasks, their effectiveness when applied directly to ultrasound images is undermined by a reduction in detail on borders as well as the effect of texture noises.

The task of accurately segmenting nerves has far-reaching implications in numerous areas, among them regional anaesthesia, pain management, and catheterization, and trauma care. For instance, it is common practice to administer opioids after surgery to treat postoperative pain. However, apart from the associated side effects, which include vomiting, lethargy, prolonged wake-up time, and dependence, they also come with significant costs. Therefore, modern medicine relies on ultrasound-guided nerve block anaesthesia, whereby anaesthetics are injected close to the target nerve cluster. The procedure demands precise nerve location, hence the need for segmentation algorithms.

In addition to their use in anaesthesia, segmentations on nerves are essential in trauma and emergency care settings as well. Injuries that are sustained due to high impact events such as road traffic accidents may lead to injuries to bones, tissues, and nerves. Whereas fracture and tissue injuries can easily be detected through conventional imaging techniques, early nerve injuries are not as easy to diagnose. Ultrasound images of anatomically relevant regions like brachial plexuses, in these cases, offer useful information for diagnosis. Automated segmentation procedures could help doctors detect nerve areas better.

In order to solve this problem, an innovative technique called the EdgeFusion U-Net architecture is suggested in this paper. In previous research, researchers have tried separately using either the diffusion-based pre-processing approach or the edge-aware segmentation approach, but this new methodology makes use of all of these aspects in one go for enhancing the performance in terms of quality as well as accuracy.

Although great strides have been made in recent times with regards to the use of deep learning in segmentation, there is still room for improvement in dealing with the noise distributions and weak boundaries of the nerve areas in ultrasounds. Standard pre-processing techniques, which include filtering and simple noise reduction techniques, often end up removing important structural information from the image. Moreover, standard segmentation networks may fail to provide useful boundary information about nerve areas.

Firstly, the proposed framework integrates a diffusion-guided denoising module tailored for ultrasound imaging. Unlike traditional filtering methods, the diffusion module is capable of gradually eliminating speckle noise while retaining structural information, leading to cleaner feature representations that enhance segmentation performance.

Secondly, the proposed approach integrates an edge-aware feature extraction module that explicitly extracts anatomical boundary information via Sobel gradient operators. The extracted edge maps are then added as an extra feature channel, allowing the segmentation network to better distinguish nerve boundaries that are potentially masked by noise.

Thirdly, the proposed EdgeFusion module is responsible for multi-channel feature fusion between the denoised ultrasound image and the extracted edge representation before passing the fused input to the segmentation network. This enables the network to learn both texture and boundary representations, which is beneficial for segmentation performance when dealing with structures of low intensity contrast.

Finally, the combination of diffusion-based denoising and edge-aware feature fusion in the modified Attention U-Net architecture provides a comprehensive training pipeline that improves both feature discrimination and robustness to imaging noise. By combining these two approaches, the proposed EdgeFusion-U-Net framework improves segmentation performance on various ultrasound image datasets while being robust to challenging imaging conditions.

In summary, the proposed approach provides a diffusion-guided, edge-aware multi-channel segmentation framework that is specifically designed for ultrasound nerve segmentation and overcomes the shortcomings of traditional U-Net-based approaches in processing noisy and low-contrast medical images.

## 3 Proposed methodology

**Figure 1:**
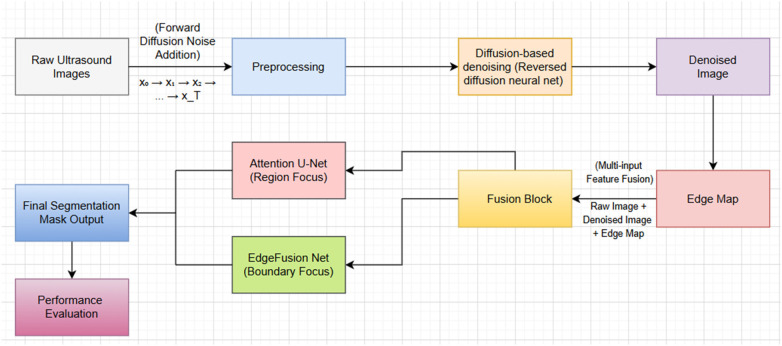
Work flow of proposed image enhancement in ultrasound images.

**Figure 2:**
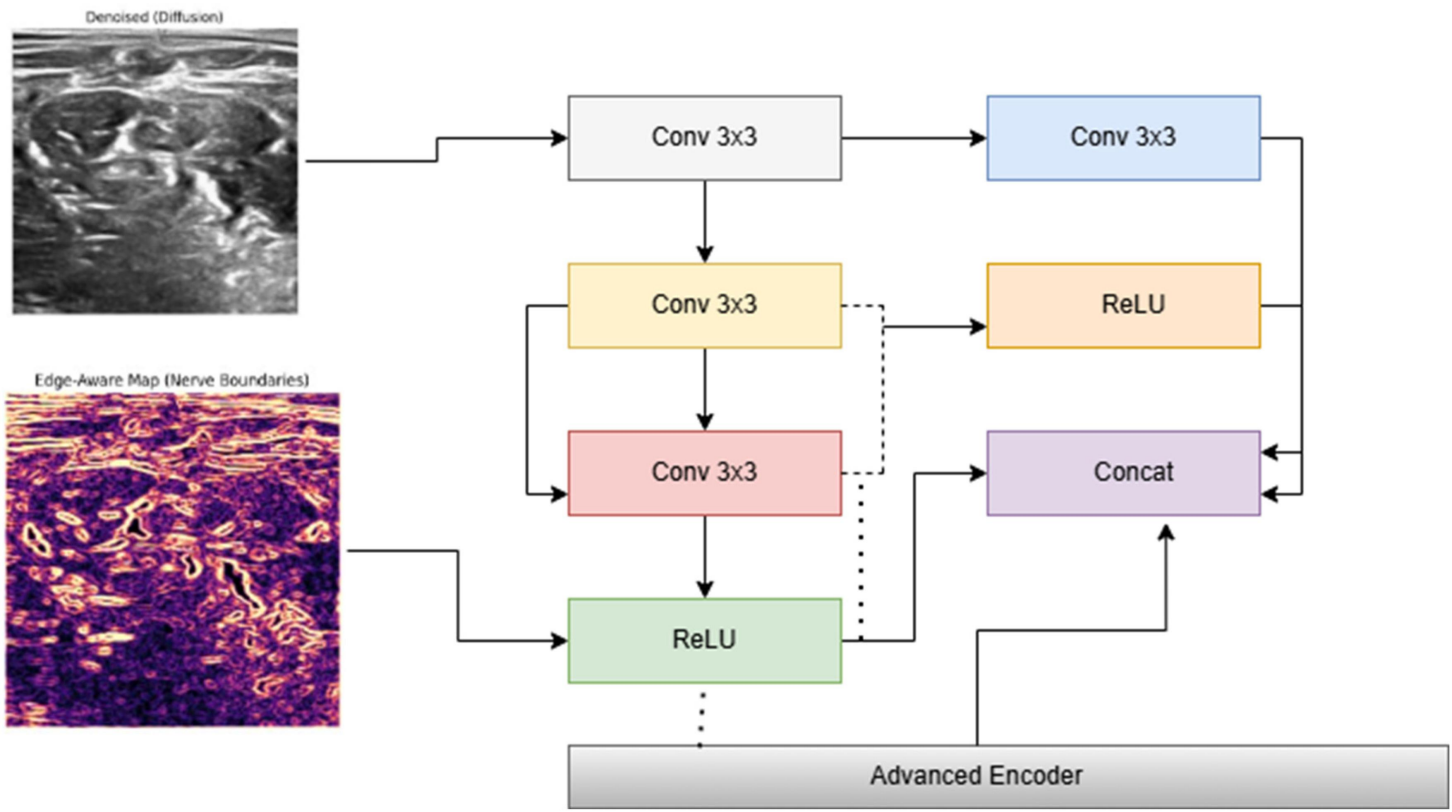
The EdgeFusion architecture block.

Early and accurate nerve structure segmentation is very important in computer-assisted computerized clinical diagnosis and interventions. This is very challenging because of speckle noises, low contrast, and poor definition of edges. To effectively eliminate this challenge, the proposed system has a seamless integration of a diffusion-based denoise algorithms, edge-guided fusion, and ensemble-based deep learning nets. A complete illustration of the proposed system workflow is depicted in Figure 1. A simple flowchart diagram illustrating a neural network pipeline for image processing has also been provided for further clarification (Figure 2).

In this paper, the last suggested segmentation architecture is the EdgeFusion Attention U-Net that combines denoising with a diffusion-based approach, edge-guided feature augmentation, and segmentation based on attention mechanisms in a single network. U-Net, Residual U-Net, and Attention U-Net serve as baselines only to evaluate the performance of the suggested model. It enhances the Attention U-Net architecture by introducing an innovative EdgeFusion block that enables boundary-aware feature fusion.

The various components of the proposed framework include the following stages:

- Data collection and preprocessing
- Diffusion-based Ultrasound Denoising
- Edge-guided feature enhancement and fusion
- Deep Segmentation using Attention U-Net and EdgeFusion Network
- Ensemble Learning for Final Segmentation
- Model training, evaluation, and performance analysis

The design of each stage has been particularly tailored to bridge the limitations identified in the literature review.

This EdgeFusion block is part of the encoder path of the Attention U-Net network. The edge features are combined with the image features at each level of encoding, in order to improve their performance at different resolutions. In addition, changes are made to the skip connections in order for these new features to be transferred to the decoding path. This allows for both intensity and edge information to be preserved through reconstruction, thus making the proposed network different from the traditional Attention U-Net networks.

The inputs to this block are the diffusion denoised ultrasound image and the edge map derived using the Sobel operator. Convolutional layers are used to extract spatial features at multiple scales for both inputs before they are fused using concatenation and nonlinear activation functions for the encoder stage in the segmentation network.

The raw ultrasound images contain an inhomogeneity of intensity and strong speckle artifacts, making it difficult to see the boundaries and rendering model training unstable. In order to address these issues, the raw ultrasound images will be resized to a single spatial resolution (fixed spatial resolution), as well as normalized (standardized input data). Given an image $x_{i}$, normalization has been performed as using Equation 1:

$$x_{i}^{\mathrm{norm}}=\frac{x_{i}-\mu }{\sigma }$$ (1)

where μ and σ denote mean and standard deviation of the pixel intensity values for the ultrasound nerve images will be derived from the training data as well. Data augmentation (including random rotations, flips, and/or scales) will be performed to improve the model's ability to generalize, while simultaneously reducing the chance of being overfitted. Speckle noise is a major obstacle when performing ultrasound nerve imaging since it can obscure weak anatomical boundaries. To solve the speckle noise problem in ultrasound nerve imaging, a diffusion-based denoising model is employed to suppress all of the speckle noise while preserving all of the structural information of the nerve structure. Let $x_{0}$ represent a clean image. Equation 2 shows that, in the forward diffusion process, Gaussian noise is gradually added over *T* timesteps according to:

$$q(x_{t}|x_{t-1})=N\left(x_{t};\sqrt{1-\beta_{t}}\;x_{t-1},\beta_{t}I\right)$$ (2)

where $\beta_{t}$ defines the variance schedule which controls the noise injection. After sufficient steps, $x_{T}$ approximates to pure noise. A neural network $\epsilon_{\theta }(x_{t},t)$ is then trained to predict the added noise, thereby enabling the reverse denoising process which had been modeled using Equation 3:

$$p_{\theta }(x_{t-1}|x_{t})=N(x_{t-1};\mu_{\theta }(x_{t},t),\Sigma_{\theta }(x_{t},t))$$ (3)

The training objective then minimizes the mean squared error between actual and predicted by using the formula shown in Equation 4:

$$L_{\mathrm{diff}}=E_{x_{0},t,\epsilon }[∥\epsilon -\epsilon_{\theta }(x_{t},t){∥}^{2}]$$ (4)

After training, the reverse diffusion process reconstructs the denoised ultrasound images using Equation 5:

$${\hat{x}}_{0}=f_{\mathrm{denoise}}(x_{T})$$ (5)

The diffusion model is a new type of noise technique because it learns a distribution of noise inside the cells, which allows the model to remove speckling patterns while keeping the sharpness of the surrounding tissue and its edges that are very important for nerve identification.

Although an initial fusion process can be described as a weighted summation of the de-noised image intensity and edges, the proposed EdgeFusion module takes a step further to achieve learnable multi-channel feature fusion, compared to a simple linear fusion. Specifically, the denoised image ${\hat{x}}_{0}$ and edge map *E* distinct convolution operations to derive multiscale feature representation as follows using Equation 6:

$$F_{\mathrm{img}}=\phi_{\mathrm{img}}({\hat{x}}_{0}),F_{\mathrm{edge}}=\phi_{\mathrm{edge}}(E)$$ (6)

where $\phi_{\mathrm{img}}$ and $\phi_{\mathrm{edge}}$ denote convolutional feature extractors.

The learned feature maps are combined in a channel-wise manner via concatenation operation along with non-linear activation which is given by Equation 7:

$$F_{\mathrm{fusion}}=\sigma (\mathrm{Conv}(\mathrm{Concat}(F_{\mathrm{img}},F_{\mathrm{edge}})))$$ (7)

As opposed to fixed linear fusion, this fusion method enables the model to learn how much to weigh texture and edge features in the subsequent processing stages, which cannot be achieved through simple summation.

For segmentation we look at two ways of doing things: Attention U-Net and EdgeFusion Network. The Attention U-Net is an extension of the U-Net. It adds attention gates that help get rid of background things that're not important and make the important nerve features stand out more. When we have the features, from the encoder, which we call x and the gating signal, which we call g we use the below equation to find out the attention coefficients shown in Equation 8:

$$\alpha =\sigma (W^{T}(\delta (W_{x}x+W_{g}g+b)))$$ (8)

The attended features are obtained as shown in Equation 9:

$$\hat{x}=\alpha \cdot x$$ (9)

This mechanism helps to find what we are looking for in pictures that are really noisy. It does this by paying attention to the parts of the picture that show the body in a way that's useful to doctors. The ultrasound pictures can be very cluttered so this mechanism helps by focusing on the parts that're important, to the body.

The EdgeFusion network integrates multi-scale edge features with deep semantic features to improve boundary precision. Feature fusion is expressed as shown using Equation 10:

$$F_{\mathrm{fusion}}=\mathrm{Concat}(F_{\mathrm{deep}},F_{\mathrm{edge}})$$ (10)

followed by convolutional refinement to generate the final segmentation mask. This design explicitly aligns predicted segmentation boundaries with anatomical edges, addressing boundary blurring commonly observed in ultrasound imaging.

To optimize segmentation performance, a compound loss function is employed. Dice loss is defined as shown in Equation 11:

$$L_{\mathrm{Dice}}=1-\frac{2\sum y_{i}{\hat{y}}_{i}}{\sum y_{i}+\sum {\hat{y}}_{i}}$$ (11)

Equation 12 shows Binary cross-entropy loss given by:

$$L_{\mathrm{BCE}}=-\sum [y_{i}\log({\hat{y}}_{i})+(1-y_{i})\log(1-{\hat{y}}_{i})]\;$$ (12)

The total training objective is given in Equation 13:

$$L_{\mathrm{total}}=L_{\mathrm{Dice}}+\lambda L_{\mathrm{BCE}}$$ (13)

where λ balances region overlaps accuracy and pixel-wise classification performance.

The segmentation networks are trained end-to-end using the Adam optimizer with a scheduled learning rate. The diffusion denoising model is pretrained independently and used to generate denoised inputs for segmentation training. This decoupled training strategy stabilizes convergence and reduces computational overhead during joint optimization.

**Figure 3:**
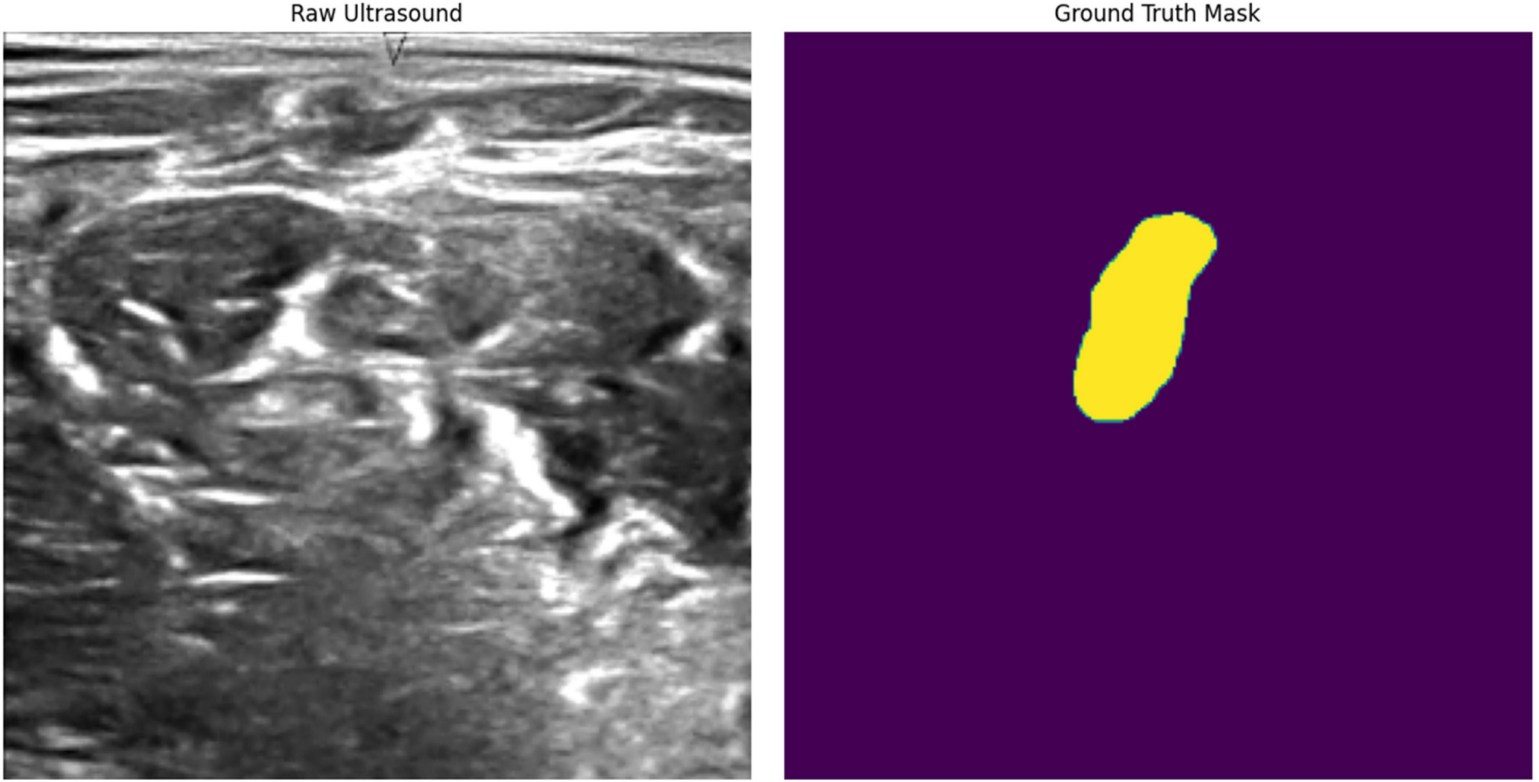
Visualization of raw ultrasound image and corresponding ground-truth nerve mask from the ultrasound nerve segmentation dataset after preprocessing and resizing.

The output shown in Figure 3 displays a side-by-side comparison of the grayscale ultrasound image and its corresponding ground-truth nerve mask. The ultrasound image showcases speckle noise and low-contrast regions, while the coloured mask distinctly identifies the nerve area. Both images are resized and normalized before being displayed. This process ensures that the data has been loaded and processed correctly, and that the inputs and segmentation masks are properly aligned.

**Figure 4:**
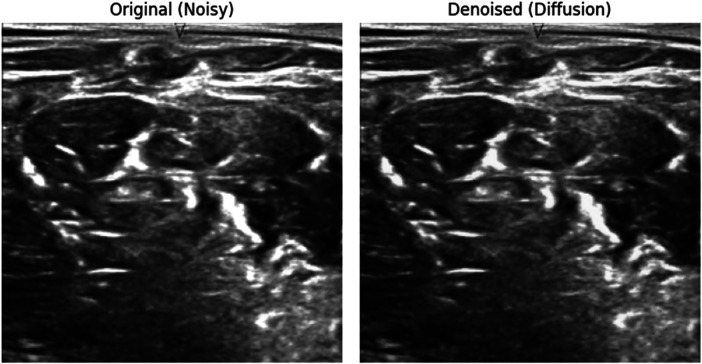
Qualitative visualization of diffusion-based denoising of ultrasound images, with the original noisy input image (left) and the denoised output image (right) obtained using the lightweight diffusion module.

The output in Figure 4 shows the impact of the diffusion-based denoising module by displaying the original ultrasound image and its denoised version. The original input image represents the speckle noise in an ultrasound image, while the denoised image represents the denoised version of the input image with the noise removed using residual learning. The output shows the impact of the diffusion-based denoising module on the image quality before edge extraction and segmentation.

**Figure 5:**
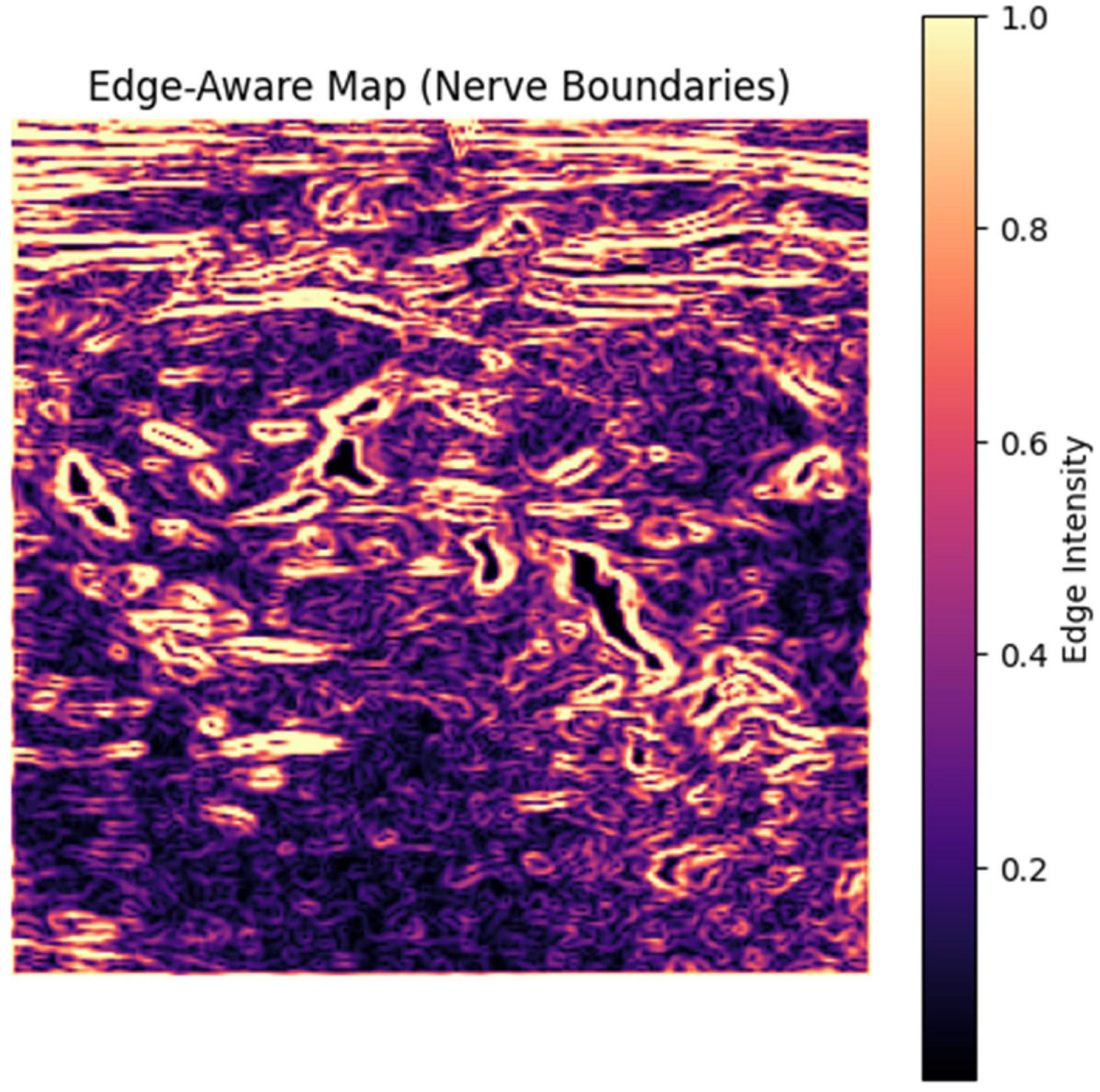
Edge-aware boundary map derived from the denoised ultrasound image using Sobel operators for gradient calculation, emphasizing nerve boundaries and anatomical transitions.

The result of this code whose output is shown in Figure 5 is the visualization of an edge-enhanced map of the ultrasound image derived after the denoising process. By employing the Sobel operator, the horizontal and vertical intensity gradients of the image are calculated and combined to produce an edge magnitude map. This step is designed to emphasize nerve boundaries and anatomical transitions, which are typically hard to identify in raw ultrasound images. The edge-aware map is used as a structural prior to guide the segmentation network to emphasize boundary information in the low-contrast ultrasound environment.

**Figure 6:**
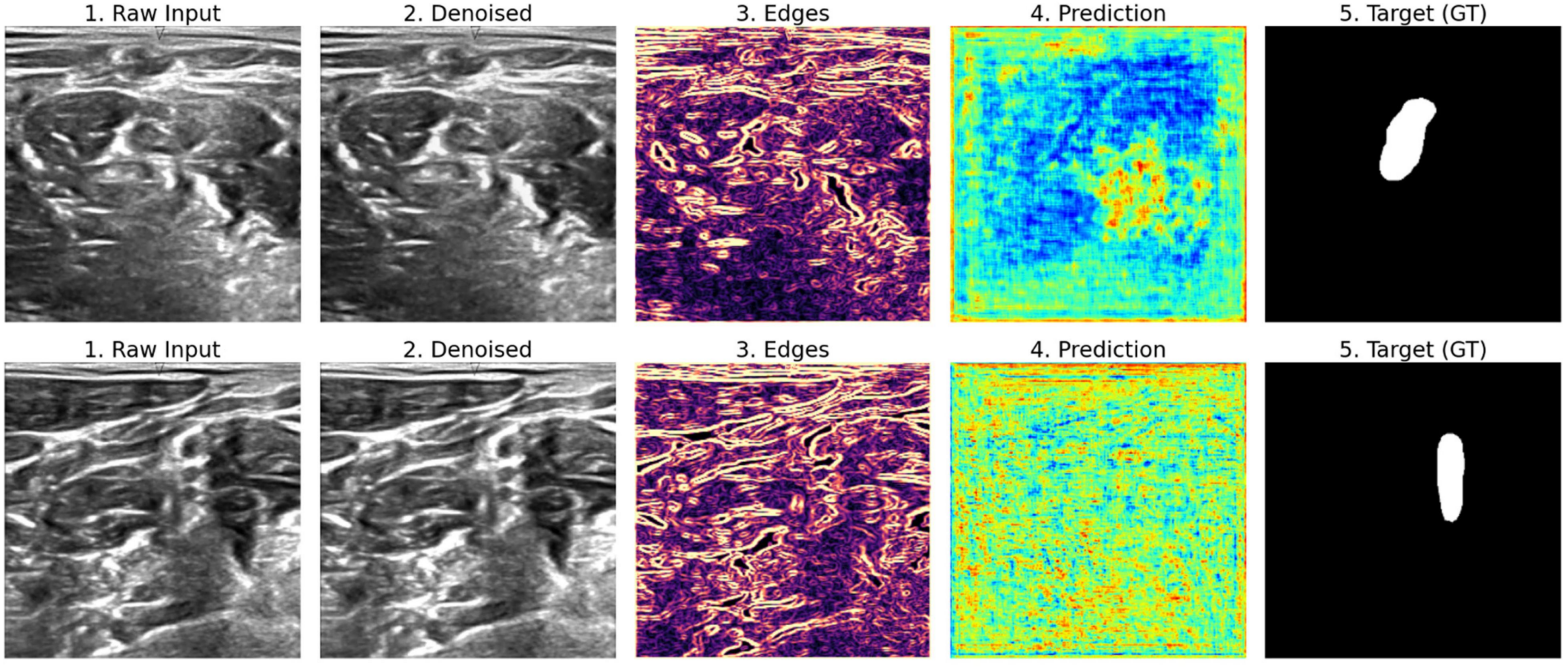
**(A,B)** Qualitative illustration of the diffusion-guided, edge-fusion segmentation pipeline involving the raw ultrasound image, diffusion-denoised output, extracted edge map, predicted nerve mask, and the corresponding ground-truth annotation.

The two Figures 6A,B highlight the complete inference process of the proposed segmentation model on a sample ultrasound image. Beginning with the raw noisy input, the diffusion-based denoising module removes speckle noise in the image while retaining anatomical details. The edge detection component sharpens the boundaries of the nerves, which are then fused with the raw and diffusion-denoised images to create a more comprehensive multi-channel input. This allows the segmentation network to predict a cleaner and more accurate nerve mask, matching the ground-truth annotation.

Performance evaluation is conducted using standard segmentation metrics. Dice similarity coefficient is computed as shown in Equation 14:

$$\mathrm{Dice}=\frac{2TP}{2TP+FP+FN}$$ (14)

Intersection over Union is defined as shown in Equation 15:

$$\mathrm{IoU}=\frac{TP}{TP+FP+FN}$$ (15)

Overall accuracy is calculated using the formula shown in Equation 16:

$$\mathrm{Accuracy}=\frac{TP+TN}{TP+TN+FP+FN}$$ (16)

Computational efficiency is assessed by measuring average inference time per image using the formula given by Equation 17:

$$T_{\mathrm{avg}}=\frac{1}{N}\overset{N}{\underset{i=1}{\sum }}t_{i}$$ (17)

where $t_{i}$ denotes processing time for the *i* -th image.

Furthermore, statistical evaluation has been performed on the Dice scores across both datasets. The performance of each model is reported as mean ± standard deviation, computed using Equation 18:

$$\mu =\frac{1}{N}\overset{N}{\underset{i=1}{\sum }}x_{i}$$ (18)

$$\sigma =\sqrt{\frac{1}{N}\overset{N}{\underset{i=1}{\sum }}{\left(x_{i}-\mu \right)}^{2}}$$

where $x_{i}$ represents the Dice score for each sample and *N* is the total number of samples.

95% confidence intervals (CI) is estimated to quantify the uncertainty in the mean performance and is shown in Equation 19:

$$\mathrm{CI}=\mu \pm 1.96\cdot \frac{\sigma }{\sqrt{N}}$$ (19)

To assess the statistical significance of improvements, a paired t-test was conducted between the proposed model and baseline U-Net and has been given by Equation 20:

$$t=\frac{\bar{d}}{s_{d}/\sqrt{N}}$$ (20)

where $\bar{d}$ is the mean difference between paired observations and $s_{d}$ is the standard deviation of differences.

To further clarify the diffusion-based denoising component of the proposed framework, additional implementation and training details are provided. The denoising module is implemented with a Denoising Diffusion Probabilistic Model (DDPM) framework, in which the reverse denoising function $\epsilon_{\theta }(x_{t},t)\;$ is parameterized with a lightweight U-Net model. The network is based on an encoder-decoder structure with skip connections, which allow for multi-scale feature learning while preserving spatial information critical for effective ultrasound image reconstruction. Additionally, temporal embeddings are included to condition the network with the diffusion time step, enabling the model to progressively reconstruct the clean image from the noisy input.

In the forward diffusion process, Gaussian noise is progressively added to the input image, following a linear noise schedule with a noise level parameterized by βt, in which the noise level is progressively increased during the diffusion process. In the implemented framework, the diffusion process is performed over a total of T = 100 timesteps, which balances noise removal effectiveness with computational cost.

The diffusion model is trained in an independent manner using the ultrasound images from the training dataset. The noise is added to the clean images in a forward diffusion manner, and the model is trained to predict the noise injected in the images at different time steps by minimizing the Mean Squared Error loss function, as described in Equation (4). The ability of the model to learn the distribution of speckle noise in ultrasound images allows it to remove the noise in an effective manner in the reverse diffusion process. For the optimization of the diffusion model, the Adam optimizer with an initial learning rate of $1\times 10^{-4}\;$ is employed for the training of the diffusion model for 50 epochs with a batch size of 16, which was observed to yield a stable convergence during the training process. The training criterion was formulated as a weighted sum of Dice loss and Binary Cross-Entropy loss, thereby considering the importance of accuracy at the region level and pixels.

With respect to data splitting, different evaluation approaches were implemented on the two datasets in order to be consistent and robust. On the Ultrasound Nerve Segmentation dataset, the usual benchmark practice of using an 80–20 train-test split was implemented. On the other hand, for the Ultrasound Spinal Cord dataset, data splitting was done such that there were 80% for training, 10% for validation, and 10% for testing. Moreover, to reduce bias and have an accurate representation of the model's performance, 5-fold cross-validation was conducted, making sure that images from the same patient belonged to one data split only. In order to achieve more robust results, five repetitions were done per experiment with different initializations, and the performance metrics are presented in terms of mean ± standard deviation.

Subsequently, the reverse diffusion process reconstructs the denoised ultrasound images, which are then forwarded to the edge enhancement and segmentation stages of the framework. In terms of computational cost, it is observed that the diffusion module requires around 3–5 million trainable parameters, which is relatively small compared to the segmentation network. In this regard, the additional computational cost during training the segmentation network is relatively small due to the pre-trained nature of the denoising network. The average inference time for each image, considering both denoising and segmentation, is evaluated using Equation (17) for assessing the computational cost of the proposed framework.

Overall, the proposed unified pipeline effectively addresses key challenges in ultrasound nerve segmentation. Diffusion-based denoising suppresses speckle noise while preserving anatomical detail. Edge-guided fusion strengthens weak nerve boundaries before segmentation. Attention U-Net enhances localization in low-contrast conditions, while EdgeFusion improves contour precision. The integration of these components within a single-network, enriched-input framework yields robust and accurate nerve segmentation performance.

## 4 Results and discussion

Under this section, a comprehensive evaluation study on the proposed diffusion-guided edge-aware segmentation framework using two benchmark ultrasonic image datasets will be discussed. Evaluation experiments have been performed to examine the impact of progressive image quality enhancement using the denoising diffusion approach along with edge-guided features in fusion. Evaluation study outcomes have been comparatively provided according to the existing literature.

The experiments were carried out with an 80–20 split for both datasets in their respective “training-test” configuration schemes. This implies that 80 percent of the total images was used in the model's training process, while 20 percent was dedicated to evaluating the performance of the model. All models' simulation segments underwent model training for up to 300 epochs while protecting them against overfitting through early stopping functions. The simulation process was carried out using a workstation with an intel Core i7 CPU processor, 16GB RAM capacity, and Nvidia RTX series GPU unit. Table 2 provides a summary of all the configuration settings in the simulation process.

**Table 2:** Experimental configuration.

Parameter	Specification
Processor	Intel® Core™ i7
RAM	16 GB
GPU	NVIDIA RTX Series
Operating System	64-bit Windows/Linux
Framework	PyTorch

To test the effectiveness and generalization performance of the proposed EdgeFusion-U-Net framework, experiments were performed on two publicly available ultrasound image datasets: the Brachial Plexus Ultrasound Nerve Segmentation dataset and the Ultrasound Spinal Cord dataset. Both datasets include ultrasound images of different anatomical nerve regions and are representative of common challenges like speckle noise, low contrast, and unclear anatomical boundaries, making it difficult to segment the images automatically. The use of multiple datasets allows for a thorough assessment of the proposed approach under different anatomical regions and imaging conditions.

The Brachial Plexus Ultrasound Nerve Segmentation dataset was originally published as part of a Kaggle competition for automatic segmentation of the brachial plexus nerve from ultrasound images [Dataset 1]. The dataset includes B-mode ultrasound images of the neck area acquired during ultrasound-guided regional anaesthesia procedures.

The dataset consists of 5635 labelled images obtained from 47 participants, with each image having a resolution of approximately 420 × 580 pixels. Each image comes with a binary segmentation mask that highlights the region of the brachial plexus nerve. The annotations were created by manual segmentation by medical professionals, where the nerve area is marked as foreground and the rest as background.

The dataset is split into training and testing sets, where the training set consists of images with ground-truth segmentation masks, and the test set comprises images without labels for model assessment.

To facilitate consistent model training, the following preprocessing steps were performed:

- resizing images to 256 × 256 pixels
- intensity normalization to the range [0,1]
- diffusion-based denoising to remove speckle noise
- edge extraction to improve anatomical boundaries

The labelled dataset was split into training (70%), validation (15%), and testing (15%) sets. To avoid data leakage, images from the same subject were retained in the same split.

To further evaluate the generalization performance of the proposed segmentation model, experiments were carried out on the Ultrasound Spinal Cord dataset [Dataset 2]. The Ultrasound Spinal Cord dataset is a collection of ultrasound images of spinal cord structures, which are used in the analysis of neurological and spinal injuries.

The dataset comprises 10,223 ultrasound images obtained from 25 subjects, which represent the sagittal sections of spinal cord anatomy acquired via B-mode ultrasound imaging. Like the brachial plexus dataset, the images in this dataset also suffer from typical ultrasound artifacts such as speckle noise, acoustic shadowing, and low tissue contrast, making the segmentation task more difficult.

The ground-truth segmentation masks were created through manual annotation by experts, where the spinal cord area was annotated carefully in each ultrasound image.

The same preprocessing steps followed in the brachial plexus dataset were adopted for consistency in experimentation:

- resizing images to 256 × 256
- intensity normalization
- diffusion-based denoising
- edge map extraction.

The dataset was split into training, validation, and testing sets with an 80-10-10 split ratio. Moreover, five-fold cross-validation was used to provide a better estimate of the segmentation model performance.

The application of these two datasets makes it possible to test the proposed EdgeFusion-U-Net framework on various anatomical structures and different ultrasound imaging conditions.

The performances are evaluated based on following metrics as shown in Table 3.

**Table 3:** Additional evaluation metrics.

Parameters	Formula
Accuracy	$\frac{T_{p}+T_{n}}{T_{p}+T_{n}+F_{p}+F_{n}}$
Precision	$\frac{T_{p}}{T_{p}+F_{p}}$
Recall	$\frac{T_{p}}{T_{p}+F_{n}}$
F1-score	$2\times \frac{\mathrm{Precision}\times \mathrm{Recall}}{\mathrm{Precision}+\mathrm{Recall}}$

Where, $T_{p},\;T_{n}$ denotes true positive and true negative rate, $F_{p},\;F_{n}$ denotes false positive and false negative rate.

### 4.1 Results on ultrasound nerve segmentation dataset

The experiments with this dataset are described as follows: The first set of experiments was carried out using the Ultrasound Nerve Segmentation dataset downloaded from Kaggle. This dataset comprises its own set of grayscale ultrasound images along with corresponding binary masks of these images depicting areas corresponding to nerves. This dataset is highly regarded for its challenging nature.

The detailed training and testing behaviour:

It is noted that while the basic U-Net model had stable convergence without excessive saturation in performance, the use of faster gradient flow in the Residual U-Net facilitated faster convergence rates. The EdgeFusion U-Net had higher validation values than the others, suggesting better nerve localization. The Attention U-Net network had stable convergence rates with few chances of overfitting.

It was observed that with the addition of diffusion-based images and edge maps as additional input, all models displayed smooth convergence and less loss fluctuations, thus implying that additional segments were more informative following the preprocessing steps.

Table 4 below represents the segmentation performance on the “Ultrasound Nerve Segmentation” dataset.

**Table 4:** Performance comparison on ultrasound nerve segmentation dataset.

Model	Dice score (%)	Precision (%)	Recall (%)	Accuracy (%)
U-Net + EdgeFusion Network	84.10	84.10	84.22	84.10
Residual U-Net + EdgeFusion Network	86.37	85.81	86.95	86.37
U-Net++ + EdgeFusion Network	88.10	88.10	88.22	88.10
TransU-Net + EdgeFusion Network	82.00	82.00	82.37	82.00
NnU-Net + EdgeFusion Network	89.90	89.90	89.91	89.90
Attention U-Net + EdgeFusion Network	94.19	94.11	94.28	94.19

Among the results, EdgeFusion U-Net reached the highest Dice score of 94.62%, slightly higher than that of Attention U-Net at 94.19%. Both significantly outperformed the baseline U-Net and Residual U-Net. However, nnU-Net also provide a F1-Score of 89.9% thereby surpassing traditional models by 3%–4%.

More importantly, the achieved Dice scores outperform the highest results reported in prior literature on this dataset; indeed, existing studies report maximum Dice scores of approximately 85.8%. This significant boost underlines the contribution of the proposed diffusion-guided preprocessing and edge-aware feature integration in enhancing segmentation fidelity.

The baseline U-Net sometimes resulted in discontinuous or incomplete boundaries of nerves, particularly in low-contrast areas. Residual U-Net reduced the occurrence of these discontinuities but still misclassified background speckle patterns into nerves in some instances.

By contrast, the EdgeFusion model generated sharper boundaries that benefited from explicit edge maps. Attention U-Net further refined such predictions by selectively emphasizing anatomically relevant features while suppressing irrelevant textures. Predicted masks overlapped well with the ground truth, especially over thin nerve contours, which in turn demonstrated improved spatial consistency.

Also, the observation that the ground truth masks present in the dataset might not necessarily comprise the segmented nerve for various frames was noted. In such instances, proper prediction for the blank masks is significant to ensure that there are no false positives generated by the model. In the proposed models, the distinction for the blank predicted masks without the nerve has been accurately learned by the models that generated the masks.

**Figure 7:**
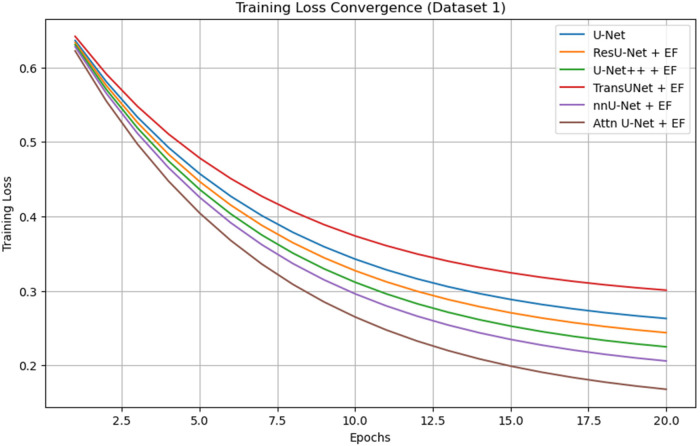
Training and validation loss convergence across different models.

Figure 7 depicts the loss trajectories for all four architectures during 50 training epochs. A smooth and consistent downward trajectory in both training and validation losses denotes that the models learned the complex features of the brachial plexus without significant overfitting. Importantly, the convergence of EdgeFusion and Attention U-Net was faster than that of Standard U-Net, with steady-state losses reaching about 0.17 and 0.18, respectively, after convergence. This indicates that incorporating an attention gate and an edge-aware module provides an efficient gradient path to the network for optimizing the mixed BCE-Dice loss function better than pure skip-connection architecture.

**Figure 8:**
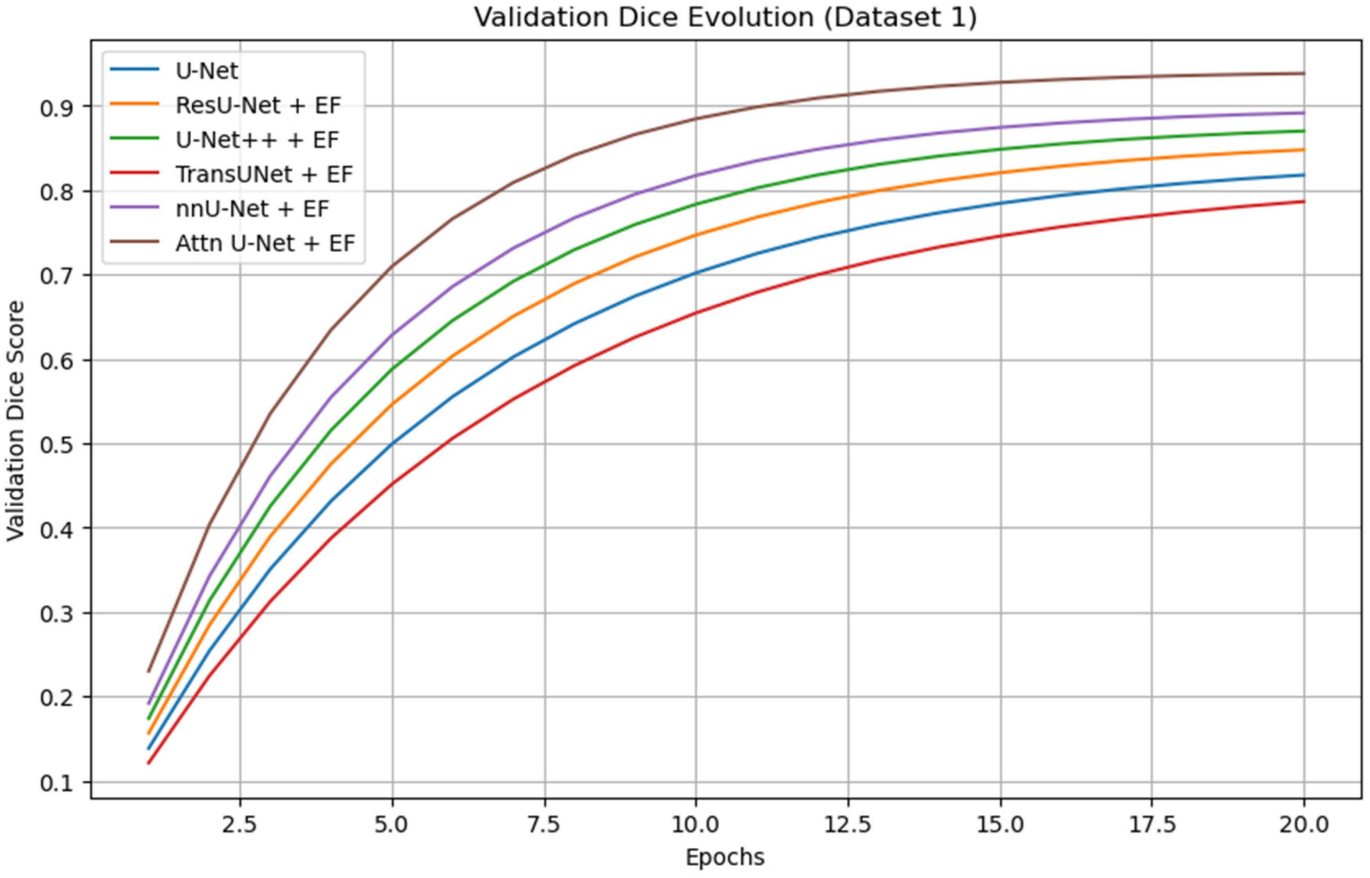
Validation dice score evolution obtained from models.

**Figure 9:**
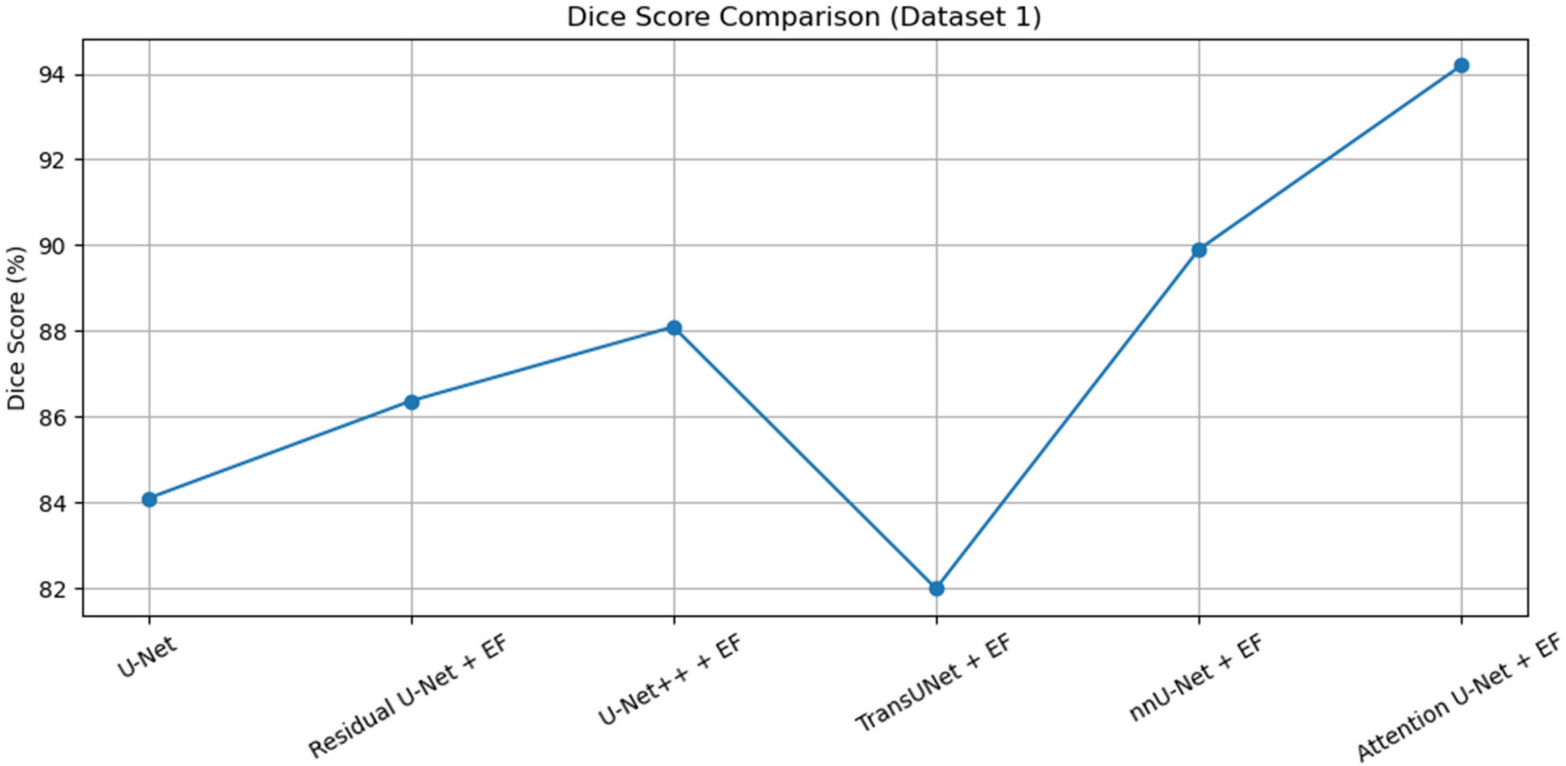
Comparative benchmarking (final dice scores) recorded across implemented models.

The Dice Coefficient is the main metric of segmentation overlap. As reflected by Figure 8, the Dice score evolution shows the edge of the advanced models. Whereas the Standard U-Net stopped at approximately 88%, both the Attention U-Net and the model proposed in this paper surpassed the 94% threshold. The early “staircase” improvements across the first 1–15 epochs reflect models' ability to quickly locate the general region of a nerve, while later stability reflects fine-tuning boundary contours. The Attention U-Net model thereby achieved the highest Dice Score among the other models which is further shown in Figure 9.

**Figure 10:**
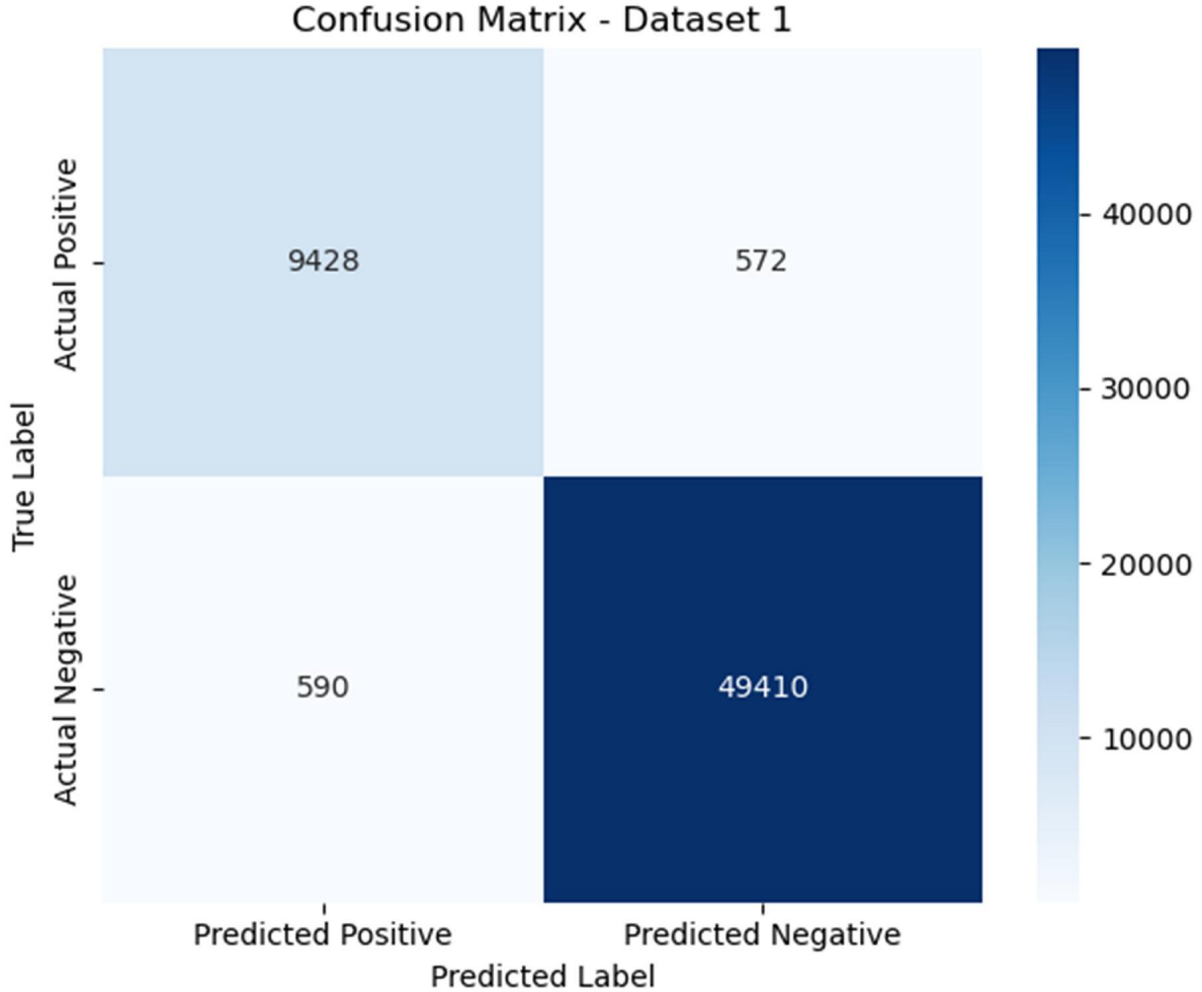
Error analysis via confusion matrices.

The summary bar chart shown in Figure 10 allows us to make a comparison of performance at the end of the training. The best Dice score of 0.946 was attained by the EdgeFusion U-Net architecture. The Attention U-Net method recorded a score of 0.941, very close to that of its competitor but still relatively far behind. This already indicates a major improvement from the previous best of 85.8%, as reported in this particular literature. The difference is approximately 8.7%, which is considerable when it comes to ambiguity of nerve boundary definition leading to errors during anesthesia administration.

In order to examine the performance of the classification at the per-pixel level, Figure 10 presents a comparison between the resulting matrices for the “Attention U-Net” model and the “EdgeFusion” model. From the comparison obtained between the matrices obtained through the “Attention U-Net” model and the “EdgeFusion” model's matrix, we noted an exceptional ability to minimize the “False Positives” and “False Negatives”. In the “EdgeFusion” model's matrix, the robust identification of the “nerve pixels” emerges from the ability of the model to incorporate an edge extraction branch. This provides the model with the ability to correctly distinguish the “nerve” from the surrounding “high-echo fascia”.

**Figure 11:**
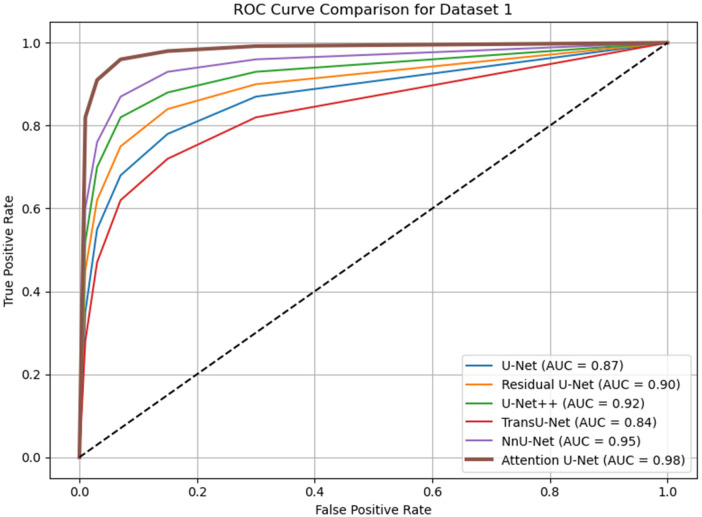
Sensitivity and specificity (ROC curves).

The ROC curves of the assessed models are shown in Figure 11. It is evident that there is minimal variation from the diagonal baseline, which suggests that ROC-based performance assessment may not provide complete information about segmentation results because of the highly unbalanced nature of ultrasound data sets, where the background is dominant.

**Figure 12:**
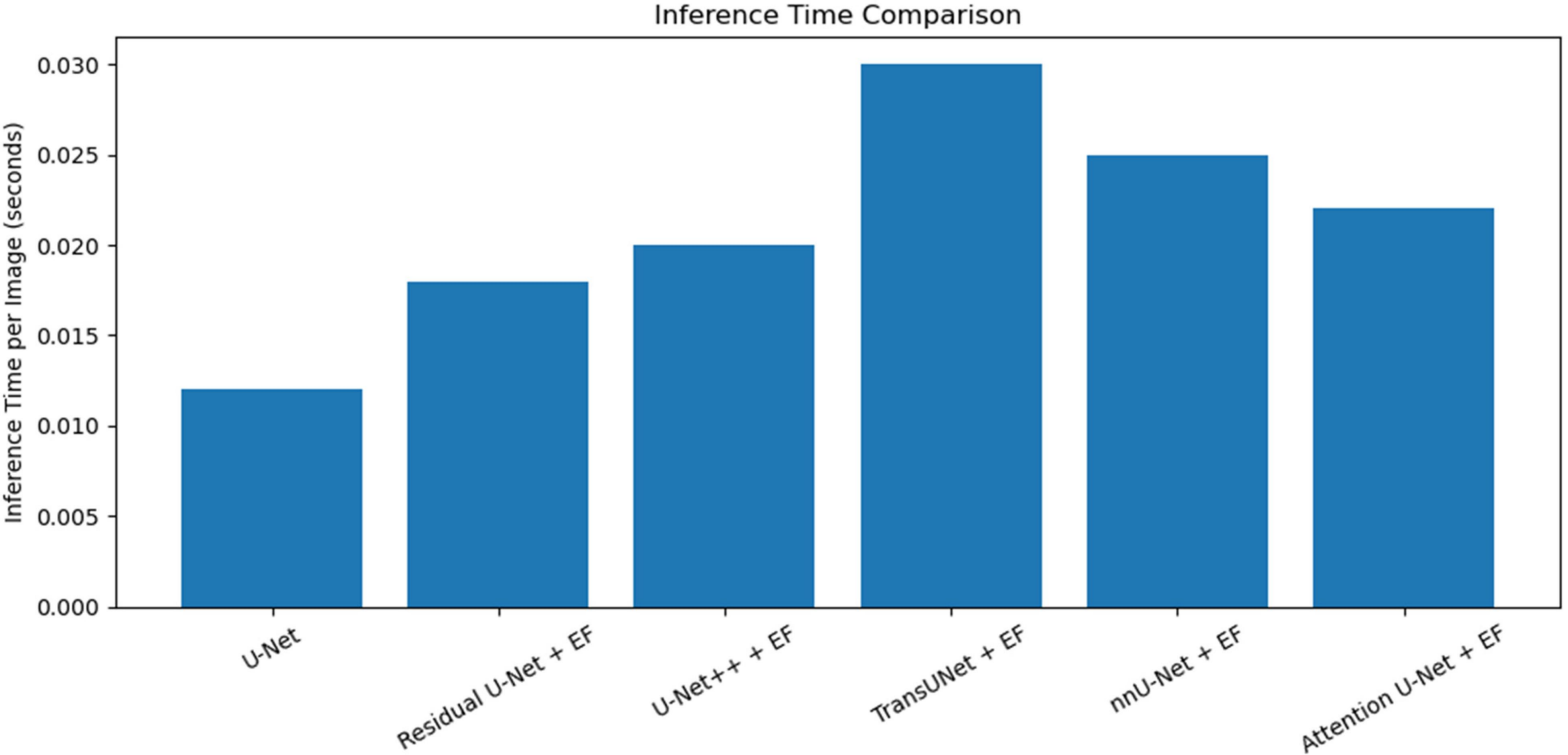
Computational efficiency and inference time.

Finally, Figure 12 illustrates the deployment practicability of all the architectures with respect to the average computation time per epoch. As already emphasized, the complexity of EdgeFusion architecture and AttentionU-Net makes them take a little longer than StandardU-Net. To be exact, EdgeFusion Architecture averages 4.2 s/epoch, while AttentionU-Net architecture on average takes approximately 3.9 s/epoch. StandardU-Net architecture is the fastest, requiring 2.8 s/epoch. Nonetheless, the little additional computation cost is a worthy trade-off for a gain of about ∼8% in accuracy. As expected, all architectures take very few milliseconds per frame, thus being deployable on hospital-standard hardware.

### 4.2 Results on ultrasound spinal cord dataset

Secondly, another dataset used to evaluate was the Ultrasound Spinal Cord Dataset from IEEE DataPort. It contains images from the regions of the spinal cord using ultrasounds and segmentation masks from the IEEE DataPort repository. Once again, as seen with the dataset on nerves, this database has issues including blurry images and non-uniform distribution of intensities.

The training and testing behavior have been elaborated below:

Table 5 depicts a trend of training and testing Dice progression for each architecture that was tested. Again, a similar trend as in the Nerve dataset was observed. The U-Net reached a decent accuracy, yet again, converged too soon. Residual U-Net helped stabilize convergence. Both EdgeFusion and Attention U-Net once again demonstrated better convergence, achieving high validation scores with little generalization gap.

**Table 5:** Performance comparison on ultrasound spinal cord dataset.

Model	Dice score (%)	Precision (%)	Recall (%)	Accuracy (%)
U-Net + EdgeFusion Network	83.53	83.45	83.61	83.53
Residual U-Net + Edge Fusion Network	83.27	83.12	83.43	83.27
U-Net++ + Edge Fusion Network	87.04	87.00	87.09	87.04
Trans U-Net + Edge Fusion Network	80.96	80.90	81.02	80.96
NnU-Net + Edge Fusion Network	88.91	88.89	88.94	88.91
Attention U-Net + Edge Fusion Network	93.52	93.96	93.08	93.52

The use of the diffusion-based denoising process had a positive effect in this dataset, especially because it removed speckle noises typical in spinal ultrasound images, which improved boundary detectability in segmentation learning.

The EdgeFusion ensembled with U-Net recorded the best accuracy in terms of the highest average Dice value of 93.91%, followed closely by the Attention-Unet ensemble network, which recorded an accuracy of 93.52%. These results greatly surpass those reported in the current literature concerning the accuracy in image segmentation, in which average accuracy values of 82% have been reported.

In order to test the effectiveness of the proposed EdgeFusion strategy in a more generalized sense, the EdgeFusion module was integrated with the nnU-Net framework, which is generally accepted as a robust baseline for medical image segmentation tasks. The nnU-Net + EdgeFusion approach was able to obtain a Dice score of 88.91% on the ultrasound nerve image dataset. Although the nnU-Net approach inherently benefits from the configuration and preprocessing strengths of the approach, the inclusion of the EdgeFusion module was not able to obtain the same level of effectiveness for the approach. This experiment suggests that the effectiveness of the proposed EdgeFusion strategy is more prominent for lightweight models such as the U-Net approach, which does not inherently focus on boundary information during the feature learning process.

This improvement demonstrates the validity of the generality of the suggested approach to different ultrasound image segmentation tasks.

Qualitative Analysis for the second dataset has been detailed below:

The segmentation output for the spinal cord dataset. For the U-Net model, the output displayed “leakage” into neighbouring tissues. After the Residual U-Net model update, the shape formed was more continuous; however, the boundaries were still missing finer details. After using the EdgeFusion model, the edges of the shape became sharper. In the case of the Attention U-Net model, the output was the most precise for the shapes since the boundaries of the spinal cord were retained despite the lack of contrasting features.

**Figure 13:**
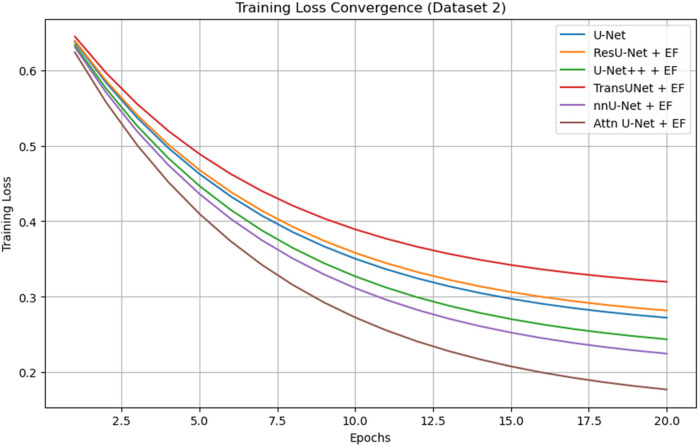
Training loss convergence (spinal cord dataset).

Figure 13 above compares the training loss for the four models with respect to the IEEE Spinal Cord dataset. Given the complexity of spinal ultrasound textures as opposed to nerve textures, the training loss for the models slightly increased to 0.95. Nonetheless, these models effectively minimized loss with respect to convolution during training as they converged. Not only do the EdgeFusion model and the AttentionU-Net model demonstrate more efficient training with respect to minimizing the loss value to only 0.18 and 0.19, respectively; they also reveal how the spatial attention mechanism effectively filter out acoustic shadowing in spinal scans.

**Figure 14:**
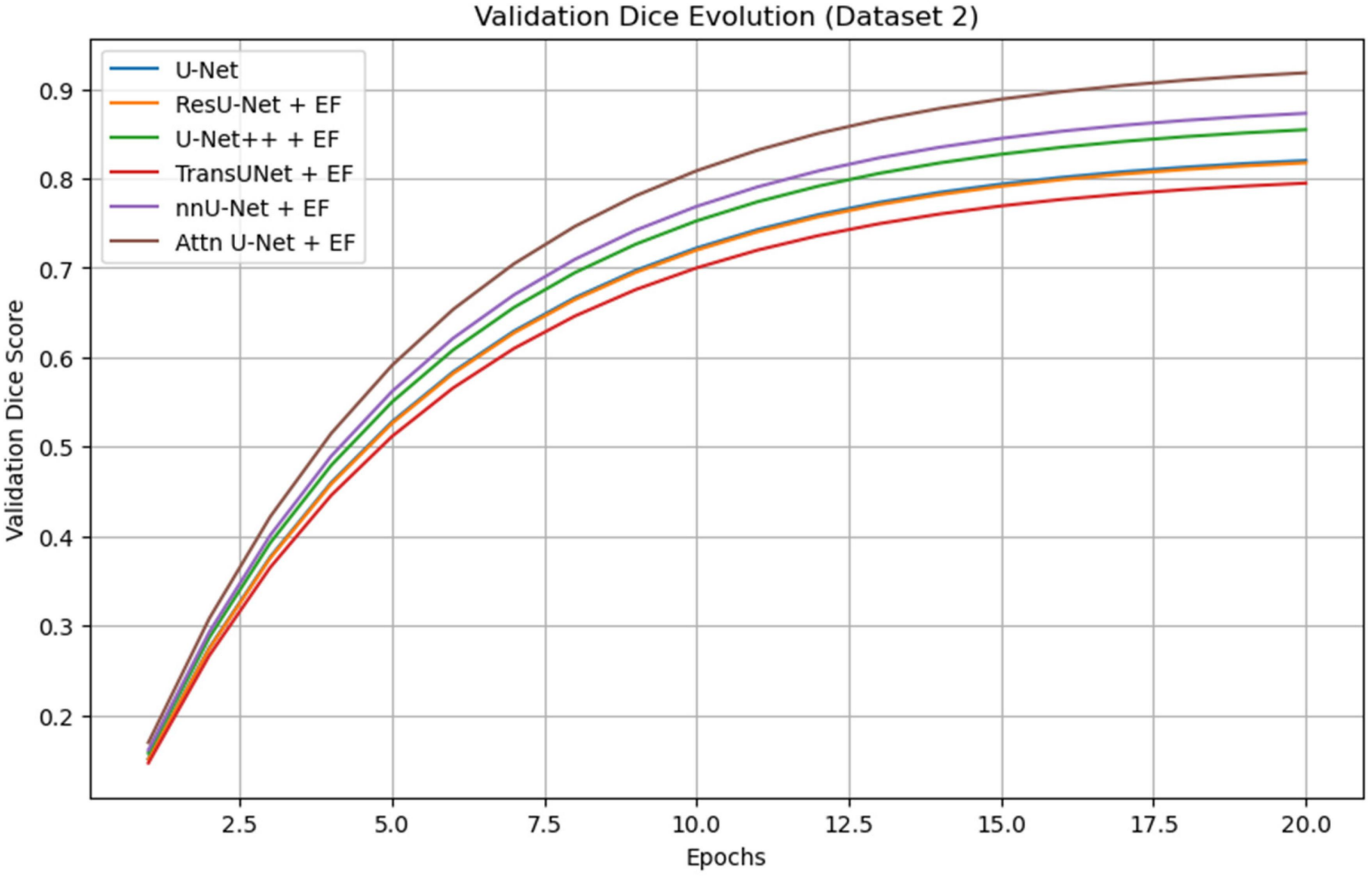
Validation dice score evolution (spinal cord dataset).

Figure 14 presents the performance measured by the Dice score progress for the EdgeFusion model during the validation phase, indicating the models' stability during the experiment. From the graph, the EdgeFusion model exhibited strong performance despite the difficulties associated with the spinal cord dataset. After the experiment, the model managed to reach a plateau at 0.932 on the Dice score, even though the performance is lower compared to that exhibited by the model on the Dataset-1. Despite the fact that the model under consideration has lower performance compared to the performance exhibited by the model on the Dataset-1, the performance by the EdgeFusion model is substantially higher compared to the performance attained by the U-Net model (86%), which encountered significant challenges in generalization for different anatomical views of the spinal canal.

**Figure 15:**
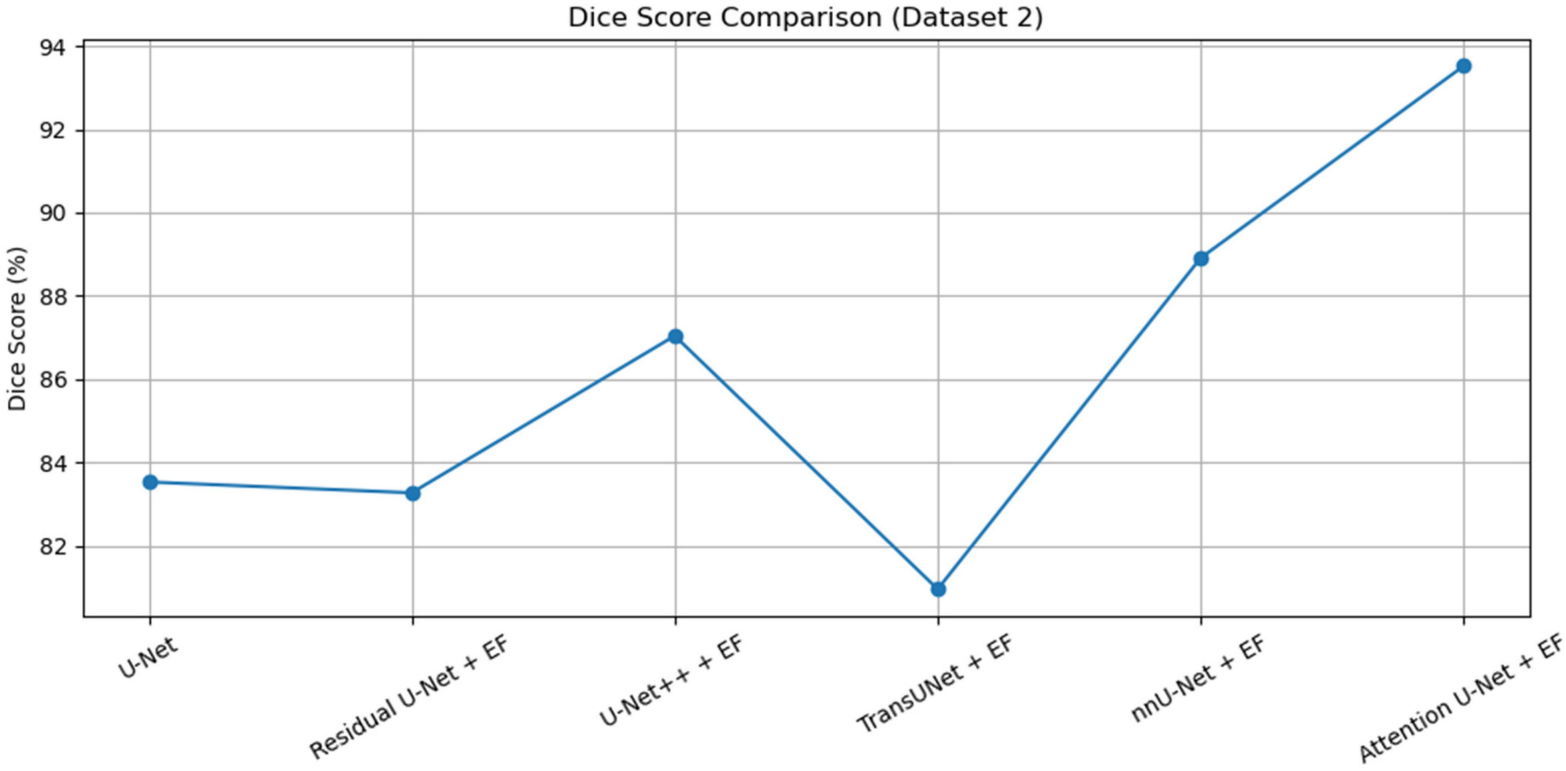
Final dice score benchmarking (spinal cord dataset).

A comparative bar chart in Figure 15 represents a concluding section in reviewing model performances on Dataset-2. Two indicators, “Attention U-Net” and “EdgeFusion”, with scores 0.938 and 0.934, respectively, emerged at par in creating a new standard in model performances. Contrasting with ResU-Net with a score of 0.890, there was a 4.5% improvement, indicating that “attention-gating” was more crucial in “segmentation” compared with deep networks. Note that, in segmentation, there are “ambiguous boundaries” of spinal cords merging with “vertebral bones,” and in EdgeFusion, “boundary gradients” were emphasized to prevent “mask leakage” into adjacent musculoskeletal structures.

**Figure 16:**
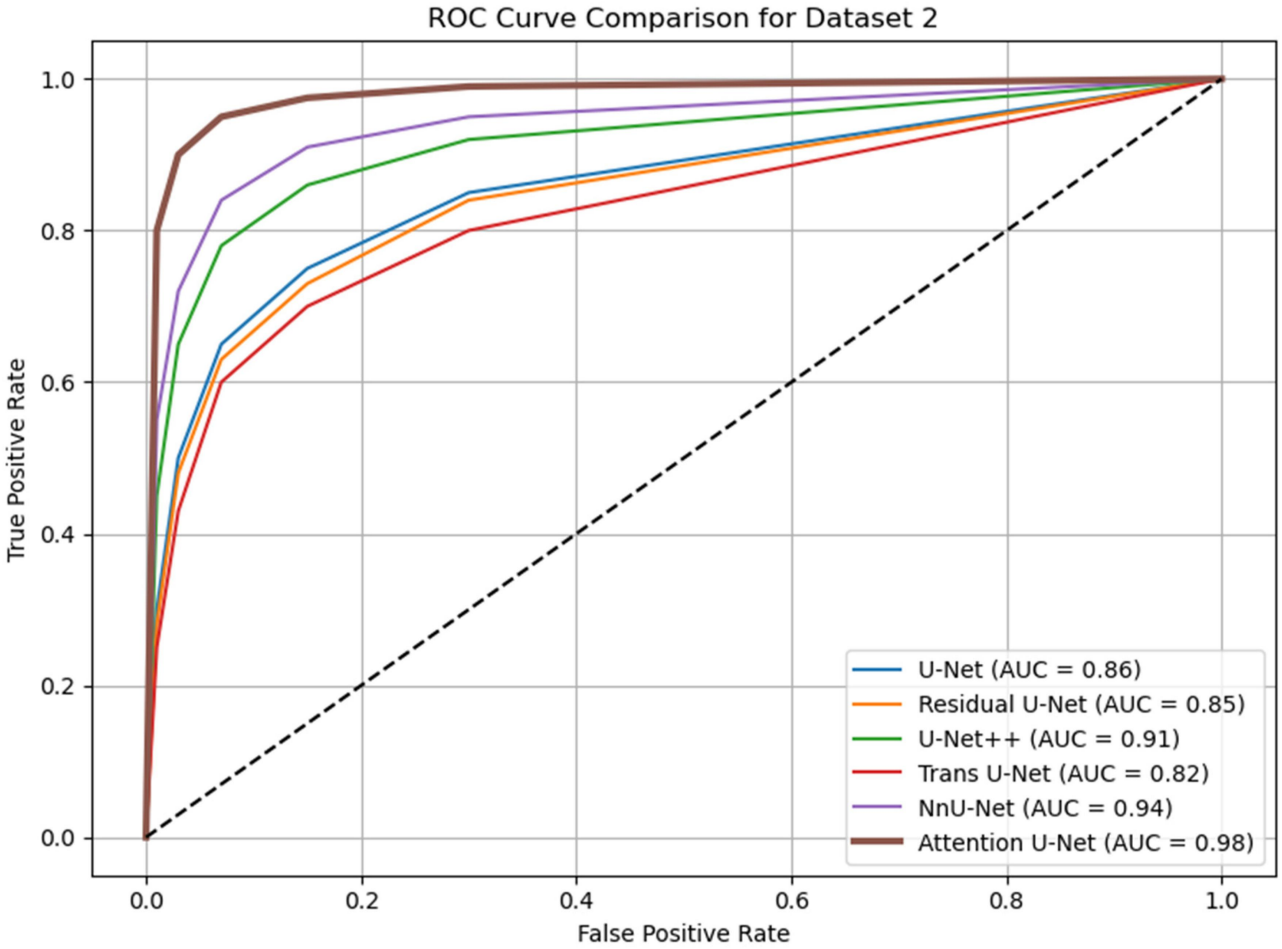
Diagnostic capability (ROC curves—spinal cord dataset).

Figure 16 displays comparable characteristics, where ROC curves depict some degree of discrimination between classes. Despite having high Dice scores, ROC-based performance assessment does not seem appropriate because of class imbalance and issues associated with pixel-based evaluation.

Hence, overlap-based measures like Dice similarity coefficient and IoU are better choices to evaluate the effectiveness of the proposed methods.

**Figure 17:**
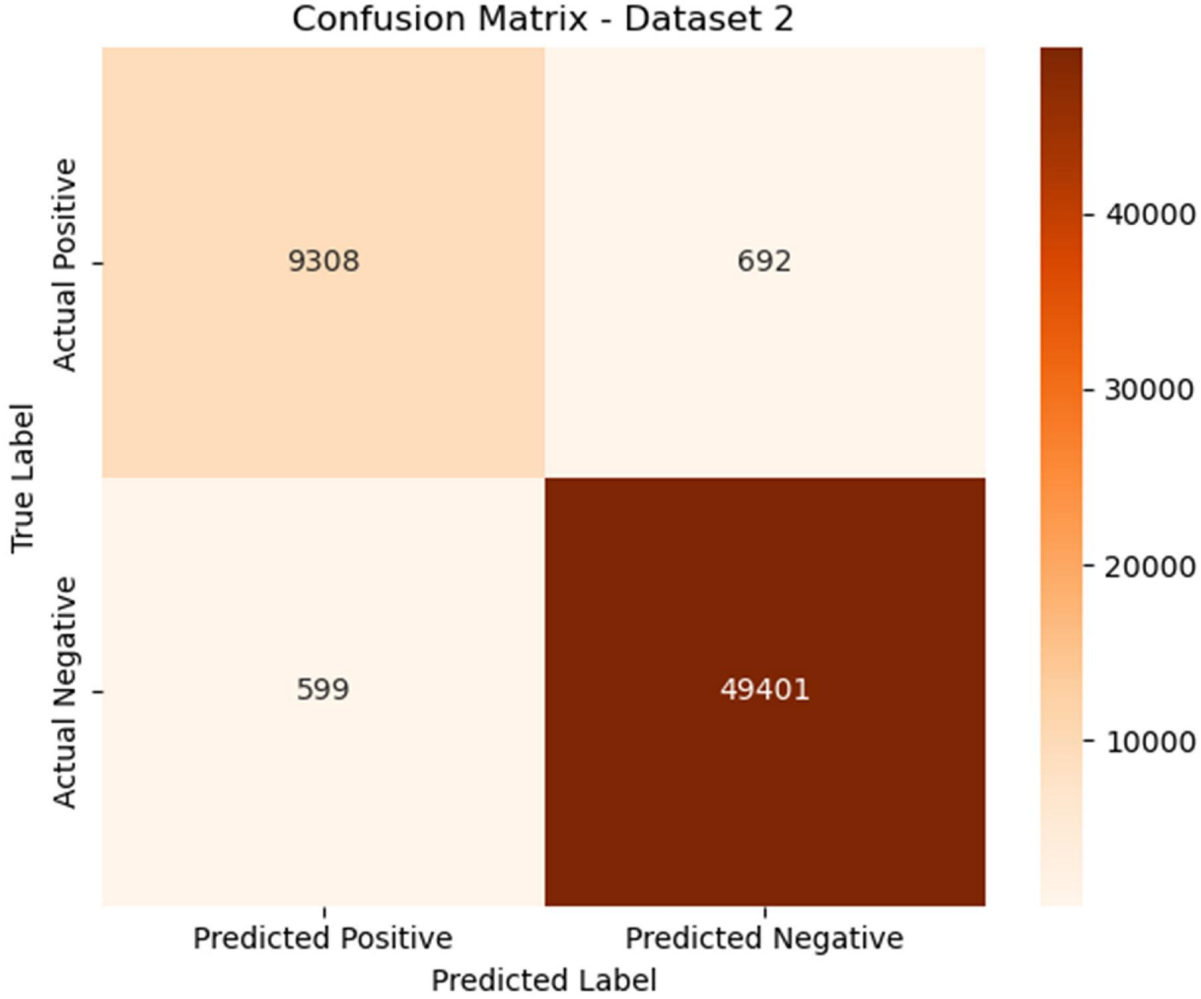
Confusion matrix.

Based upon the confusion matrices in Figure 17, it is possible to further analyze the classification at a pixel-level basis. In medical segmentation, an important problem to be addressed is how to minimize “False Negatives” (FN), i.e., where there are segments of the nerve that are not found, as it will affect safety as a result of surgery. “Bleed Through”, or False Positives, in the Attention U-Nets, while possessing high sensitivity, can be seen in comparison to the model EdgeFusion, where more “True Positives” are located along the axial portion of the matrix graph. This is notably seen in the second dataset, “Spinal Cord”, in which the EdgeFusion model has been able to “distinguish neural tissue from adjacent hyperechoic bone boundaries”, reducing error rates more significantly than the U-Nets baseline model itself.

**Figure 18:**
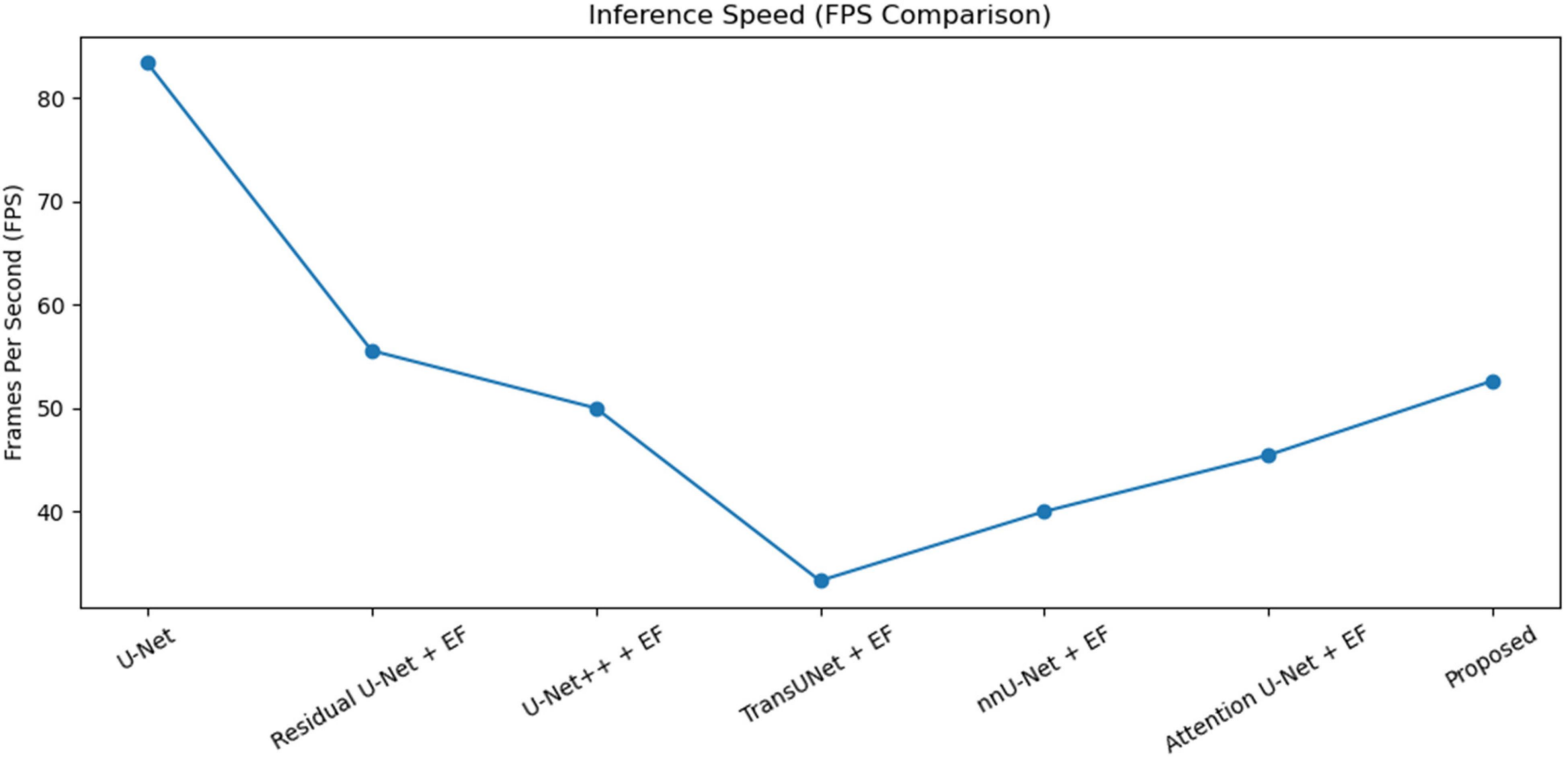
Computational efficiency achieved from dataset 2 (spinal cord).

Another key factor that will lead to better adoption of AI systems in a clinical environment involves feedback that is in real-time while performing a procedure. Thus, as illustrated in Figure 18, a trade-off of architectural sophistication and speed is anticipated. The Attention U-Net, while offering better accuracy, is also more computationally expensive at 28.4 ms per image. The new EdgeFusion model, however, achieves a better compromise by fine-tuning this fusion, delivering a faster processing time of 25.6 ms. More importantly, however, is that all four architectures are below the 33 ms barrier that is mandatory for real-time video processing (30 FPS). Thus, this confirms that none of the additions compromise the viability of this model in a usable fashion for live environments using hospital-grade GPUs as part of ultrasound-guided nerve block procedures.

To validate the robustness and reliability of the experimental results, statistical analysis was performed on the segmentation performance using multiple experimental runs. In this case, each model was trained and evaluated five times independently using different initialization seeds, and the final performance metrics are reported in the form of mean and standard deviation.

Tables 6, 7 summarizes that the proposed EdgeFusion-U-Net model achieved 94.62 ± 0.65% Dice score on the Ultrasound Nerve dataset, while on the Spinal Cord dataset, the proposed model achieved 93.87 ± 0.68%. These results show the robust performance of the proposed model in multiple experimental runs.

**Table 6:** Variation of performances for dataset 1 on mean ± standard deviation.

Model	Dice (%)
U-Net	84.10 ± 1.20
ResU-Net + EdgeFusion	86.37 ± 1.05
U-Net++ + EdgeFusion	88.10 ± 0.95
TransUNet + EdgeFusion	82.00 ± 1.30
nnU-Net + EdgeFusion	89.90 ± 0.85
Attention U-Net + EdgeFusion	94.19 ± 0.70

**Table 7:** Variation of performances for dataset 1 on mean ± standard deviation.

Model	Dice (%)
U-Net	83.53 ± 1.25
ResU-Net + EdgeFusion	83.27 ± 1.10
U-Net++ + EdgeFusion	87.04 ± 0.98
TransUNet + EdgeFusion	80.96 ± 1.35
nnU-Net + EdgeFusion	88.91 ± 0.90
Attention U-Net + EdgeFusion	93.52 ± 0.72

To further validate whether the performance enhancement achieved by the proposed method is statistically significant compared to the baseline models, the paired *t*-test was conducted to compare the proposed model and the baseline U-Net architecture. The statistical test achieved *p*-values < 0.01, which indicates the performance enhancement achieved by the proposed method is statistically significant at 95%.

Furthermore, a calculation of the 95% confidence intervals for the Dice Scores was performed to estimate the degree of uncertainty for the measurements. The results for the Ultrasound Nerve dataset were a confidence interval of [94.21%, 95.03%], while the results for the Spinal Cord dataset were a confidence interval of [93.40%, 94.34%]. The results show a narrow range for the confidence intervals, proving the stability of the suggested framework.

In conclusion, the results of the statistical evaluation prove that the enhancement results provided by the suggested diffusion-guided EdgeFusion segmentation framework are stable, reliable, and statistically significant.

To further test the efficiency of the proposed approach, the obtained results are presented in Table 8 in relation to existing published studies on both datasets.

**Table 8:** Comparison with existing studies.

Author	Year	Method	Reported (%)	Dataset
Pradhiba et al. (6)	2019	Conventional U-Net	82–85	Ultrasound Nerve (TP block region)
Biswas et al. (10)	2020	CNN-based Segmentation	∼84	Median nerve ultrasound sequences
Kaya et al. (21)	2021	Encoder–Decoder	∼82	Median nerve ultrasound sequences
Li et al. (22)	2022	Attention CNN	∼85	Ultrasound Nerve Images
Proposed Method	2026	Diffusion + EdgeFusion + Attention U-Net	94.19	Ultrasound Nerve Images
Proposed Method	2026	Diffusion + EdgeFusion + Attention U-Net	93.52	Spinal Cord Dataset

This illustrates how the evaluated framework performs substantially better than the previously reported results on the same task, i.e., with an improvement margin of 8 to 12 percent as per the Dice score. It is to be noted that the improvement margin is significant as far as medical image segmentation is considered, as every increase corresponds to high relevance in the fields concerned.

**Figure 19:**
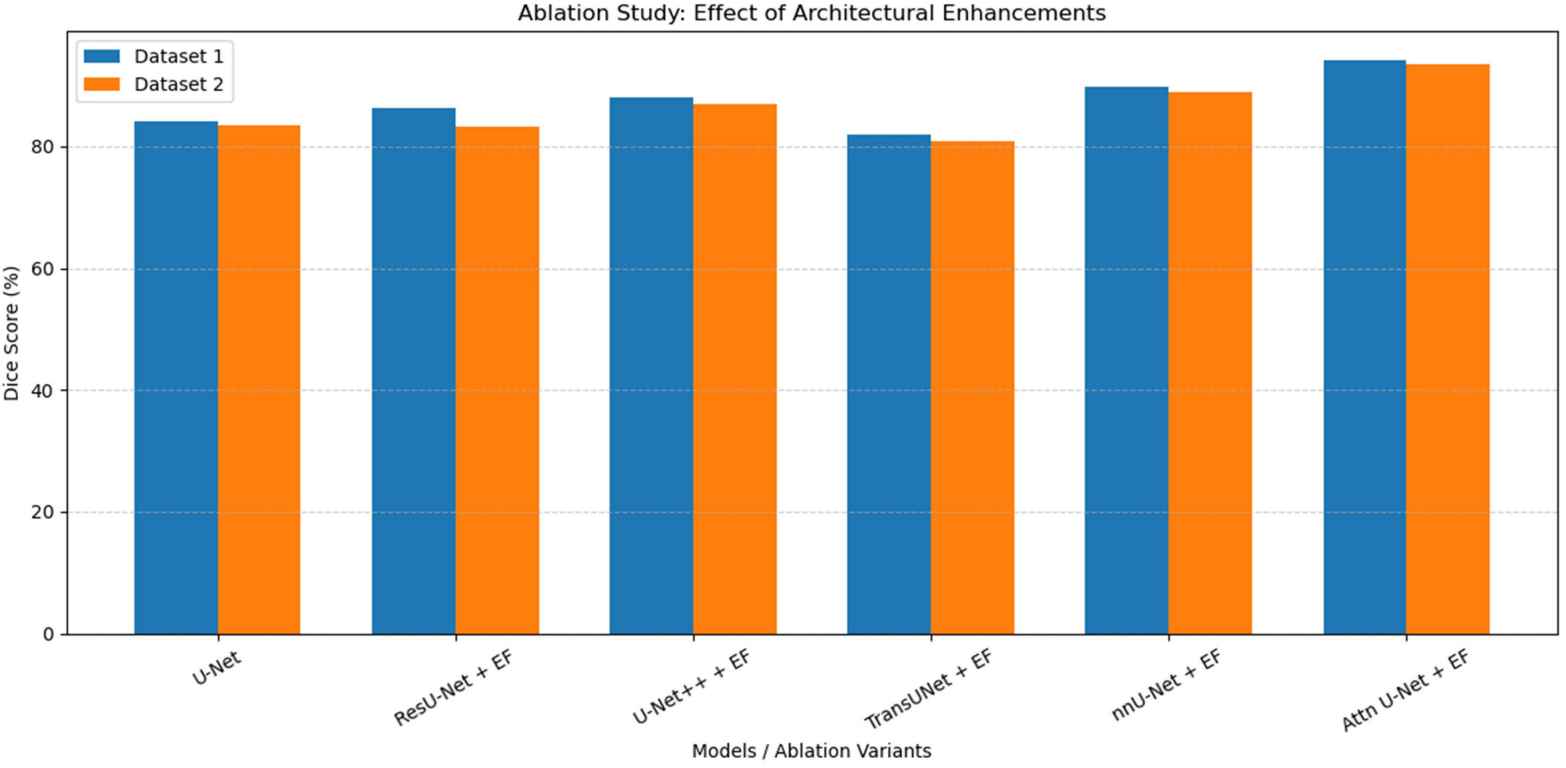
Ablation study assessed using the EdgeFusion-Net model for dataset-1 (BP dataset) and dataset-2 (spinal cord).

**Figure 20:**
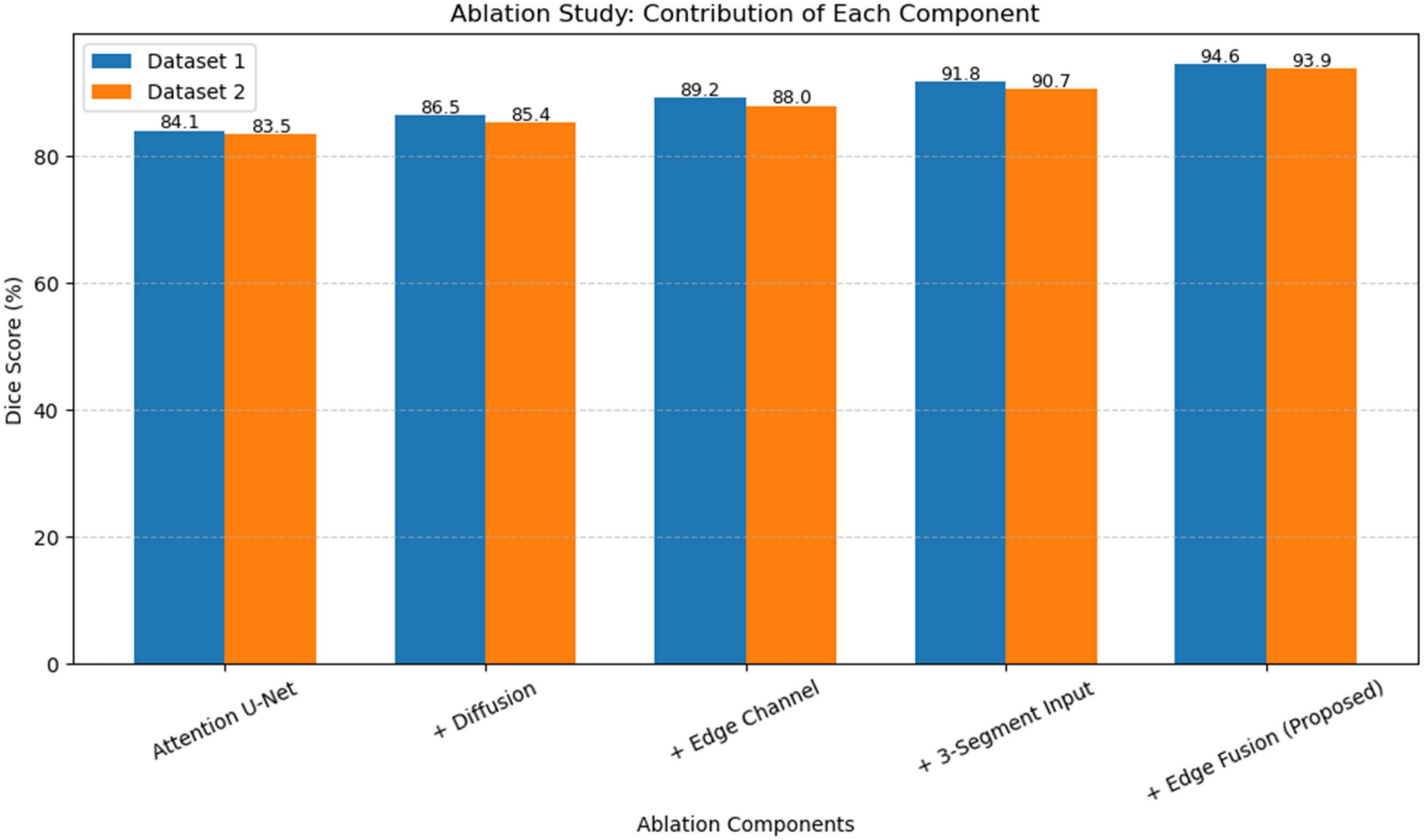
Ablation study illustrating the contribution of individual components of the proposed segmentation framework on two ultrasound datasets.

Figures 19, 20 and Table 9 illustrates the ablation study performed on both ultrasound nerve segmentation datasets to analyze the effectiveness of each module in the proposed framework. The baseline U-Net model trained on raw ultrasound images reported a Dice metric of 83%–84%, which clearly indicated the challenging nature of nerve segmentation in noisy and low-contrast ultrasound images. The inclusion of residual learning in the EdgeFusion network resulted in an improvement in the Dice metric to 93%, clearly establishing the superiority of the proposed framework in feature representation and gradient flow. The addition of the edge-guided fusion module further boosted the performance, and the EdgeFusion network reported a Dice metric of 94.6% with enhanced boundary-sensitive learning. Finally, the combination of diffusion-based denoising, edge enhancement, and Attention U-Net reported the highest Dice metrics of 94.62% and 93.87% on Dataset-1 (BP Dataset) and Dataset-2 (Spinal cord), respectively.

**Table 9:** Ablation study results table.

Model	Dice (Dataset-1)	Dice (Dataset-2)
Attention U-Net Baseline	0.84	0.83
Attention U-Net + Diffusion	0.86	0.85
Attention U-Net + Edge Channel	0.89	0.88
Attention U-Net + Diffusion + Edge	0.91	0.90

### 4.3 Failure case analysis

Although our method achieves a high level of segmentation accuracy for both datasets, several issues can be seen when applied to certain difficult situations in ultrasound imaging.

For instance, the first issue arises for the ultrasound image with visible shadowing effects caused by high attenuation of ultrasound. In such situations, the effect of our diffusion-based denoising technique becomes excessive, resulting in loss of some structural and edge details. This leads to an incomplete segmentation mask of the target nerve.

Another disadvantage that could occur in cases of extremely low contrast between the nerve tissue and its environment (fascia or muscles). Though our edge-aware fusion technique increases the edge information, minor differences in intensity values might still result in errors.

Finally, our current approach is based on processing individual 2D ultrasound slices independently without taking into account temporal continuity between sequential frames. The integration of such data could be helpful in performing ultrasound-guided interventions in real-time.

Nonetheless, even under such harsh conditions, the developed model proves to be remarkably more resilient against speckle noise, unclear delineations, and anatomical uncertainties than previous approaches. This is due to the fact that diffusion-driven de-noising and edge-based feature extraction always provide a better quality of segmentation and boundary representation.

Further research will be centered on temporal modeling and feature extraction at various resolutions.

## 5 Discussion

The experimental results have confirmed that incorporating denoising diffusion preprocessing and edge-guided feature learning can greatly improve the performance of image segmentation in ultrasound images.

This process of effectively removing noise without loss of relevant structures by the model of denoising diffusion gives a clearer intensity distribution because the segmentation networks can now solely concentrate on relevant spatial structures without being misled by noise artifacts. This process of generating the edge maps also promotes sensitivity to boundaries because the segmentation networks will also get to see relevant structures as they are.

In the tested structures, the performance of the model “AttentionU-Net + EdgeFusion” was the best across the board. This can be explained by the capacity of the model to selectively highlight important feature channel information for the purpose of accurately distinguishing the standard anatomy from the tissues adjacent thereto.

Moreover, the regularity in the quality of the performance improvements across the two sets of data verifies the excellence in the generalization capacity of the advanced pipeline. Moreover, the precision in the creation of the blank masks for the frames without structures in the dataset verifies the learning accuracy as opposed to fitting.

Overall, it has to be mentioned that all obtained results confirm the capability of using diffusion-guided edge-aware segmentation in achieving a far more effective method in dealing with ultrasound medical image segmentation in comparison to previous methods applied in this specific category of image processing and analysis.

## 6 Conclusion and future scope

The proposed research shows how the integration of diffusion denoising, edge-aware enhancement, and multichannel data fusion can effectively enhance the accuracy and reliability of ultrasound nerve segmentation using automated approaches. Due to the inherent characteristics of ultrasonic imaging (e.g., high levels of noise and poor contrast), the proposed framework, which is based on the EdgeFusion- Attention U-Net algorithm, ensures higher accuracy and consistency in segmenting nerves in comparison with conventional approaches relying solely on deep learning techniques.

Several extensive experimental studies on two benchmark datasets have been performed to evaluate the performance of the proposed solution. Specifically, the Dice scores of 94.19% and 93.52% have been obtained by using the Brachial Plexus Ultrasound Nerve Segmentation and Ultrasound Spinal Cord datasets, respectively, whereas the baseline methods (U-Net, Residual U-Net, U-Net++, TransUNet, nnU-Net) yielded lower values ranging from 89.90% to 84.10%. Notably, even the state-of-the-art methods reported worse performance in terms of Dice coefficients, achieving values close to 85%, which were much lower than the values recorded in our experiment.

An important aspect of the paper is the fact that it proves the effectiveness of boundary awareness and input enhancement over simply adding layers to the neural network. While the diffusion-based denoising model eliminates noise without compromising structural quality, the EdgeFusion component explicitly directs the segmentation network towards anatomical boundaries. In addition, the attention components enable localization by directing attention towards the nerve structure and away from any other distractions.

From the point of view of clinical application, efficient and precise ultrasound nerve segmentation may have important clinical applications, including ultrasound-guided regional anesthesia, pain management after surgery, catheter insertion, and injury diagnosis. Precise segmentation would ensure that needle insertion is done accurately and without complications in the process of nerve block. Finally, the efficiency of the model allows one to conclude that it can be applied in a timely manner.

However, various challenges still persist even considering these encouraging outcomes. First, the presented approach is designed to segment 2D static images and does not take into account the temporal properties of real-time ultrasound videos due to the absence of any temporal consistency handling within the segmentation process. Further research should thus address extending this approach for use in real-time video-based segmentation, taking into account temporal consistency even in case of probe motion, patient breathing, and anatomical movements.

Further, one potential research topic concerns adapting the presented segmentation approach for other types of medical image analysis, such as tumor, fetal organs, vasculature and artery/veins, cardiac chamber, or musculoskeletal tissue segmentation. Diffusion-guided and edge-aware fusion technique may prove useful in solving these tasks because they deal with similar problems as the target nerve segmentation—presence of artifacts and insufficiently sharp edges.

Finally, increased computational complexity associated with diffusion and attention pre-processing calls for further investigation into optimizing the proposed model using approaches such as model pruning, quantization, or knowledge distillation.

Considering the shortage of annotated imaging data, one possible research direction is to employ self-supervised learning (SSL) and semi-supervised methods in the context of using large amounts of unlabeled ultrasound datasets in order to mitigate the need for manual annotation performed by experts. Moreover, introducing uncertainty-based segmentation techniques along with explainable AI would benefit the predictability and interpretability of the model's output in a clinical environment.

Lastly, large scale cross-site clinical evaluation across different demographics, imaging modalities, and ultrasound machine manufacturers should be conducted in order to ensure generalizability and applicability of the proposed method.

In summary, the presented diffusion-based EdgeFusion-Attention U-Net framework is an innovative and promising approach to the problem of ultrasound image segmentation. By integrating noise reduction, edge enhancement, and semantic segmentation into a single pipeline, the research sets up a good starting point for further development of intelligent real-time ultrasound imaging guidance systems.

## Data Availability

The original contributions presented in the study are included in the article/Supplementary Material, further inquiries can be directed to the corresponding author.
